# Coding genomes with gapped pattern graph convolutional network

**DOI:** 10.1093/bioinformatics/btae188

**Published:** 2024-04-11

**Authors:** Ruo Han Wang, Yen Kaow Ng, Xianglilan Zhang, Jianping Wang, Shuai Cheng Li

**Affiliations:** Department of Computer Science, City University of Hong Kong Shenzhen Research Institute, Shen Zhen, 518063, China; Department of Computer Science, City University of Hong Kong, Hong Kong, 999077, China; Department of Computer Science, City University of Hong Kong Shenzhen Research Institute, Shen Zhen, 518063, China; Department of Computer Science, City University of Hong Kong, Hong Kong, 999077, China; State Key Laboratory of Pathogen and Biosecurity, Beijing Institute of Microbiology and Epidemiology, Beijing, 100071, China; Department of Computer Science, City University of Hong Kong Shenzhen Research Institute, Shen Zhen, 518063, China; Department of Computer Science, City University of Hong Kong, Hong Kong, 999077, China; Department of Computer Science, City University of Hong Kong Shenzhen Research Institute, Shen Zhen, 518063, China; Department of Computer Science, City University of Hong Kong, Hong Kong, 999077, China

## Abstract

**Motivation:**

Genome sequencing technologies reveal a huge amount of genomic sequences. Neural network-based methods can be prime candidates for retrieving insights from these sequences because of their applicability to large and diverse datasets. However, the highly variable lengths of genome sequences severely impair the presentation of sequences as input to the neural network. Genetic variations further complicate tasks that involve sequence comparison or alignment.

**Results:**

Inspired by the theory and applications of “spaced seeds,” we propose a graph representation of genome sequences called “gapped pattern graph.” These graphs can be transformed through a Graph Convolutional Network to form lower-dimensional embeddings for downstream tasks. On the basis of the gapped pattern graphs, we implemented a neural network model and demonstrated its performance on diverse tasks involving microbe and mammalian genome data. Our method consistently outperformed all the other state-of-the-art methods across various metrics on all tasks, especially for the sequences with limited homology to the training data. In addition, our model was able to identify distinct gapped pattern signatures from the sequences.

**Availability and implementation:**

The framework is available at https://github.com/deepomicslab/GCNFrame.

## 1 Introduction

Advances in next-generation sequencing technology have led to an explosive accumulation of genome sequences. Accurate characterization of those newly discovered sequences, which is inherently a problem of labeling new sequences with the knowledge from known sequences, improves our understanding of biological systems. Thus, there is a critical need for computational tools to analyze genome sequences.

The homology search serves as a primary approach to studying sequences. Recently, homology has been computed with alignment-free (AF) methods to avoid the pitfalls of sequence alignment (Zielezinski *et al.* 2017). These AF methods calculate the frequency or uniqueness of different *k*-mers to characterize genomes and use various statistical measures to determine the similarity between different genomes. However, several drawbacks limit the robustness of these AF approaches. First, approaches based solely on *k*-mer distributions forfeit all possibilities of capturing crucial information on the interaction between *k*-mers or the order of their occurrences. Second, most AF approaches require exact matches of *k*-mers, which overlook the frequent genetic variations in biological sequences, including single-nucleotide polymorphisms (SNPs) and insertions or deletions (InDel).

An alternative to AF-based input is the one-hot encoding of sequences, which is commonly used in neural network models for bio-sequence analyses. However, such encodings adapt poorly to sequences of variable length. They also have no capability to associate neighboring nucleotides into frequently occurring components (Zhang *et al.* 2019).

An additional drawback restricts all such vector-based (or matrix-based) inputs, whether one-hot or AF since they inevitably introduce an ordering over the encoded features. For instance, a vector of *k*-mers would implicitly identify each *k*-mer by a chosen position within the vector. An arbitrarily chosen ordering would result in biases in the trained neural network models. On the other hand, nonarbitrary ordering is hard to realize. For instance, one can require that neighboring elements of the vector represent *k*-mers that differ only by a single nucleotide. However, since each *k*-mer has 3*k* neighbors that differ by a single letter, this ordering cannot be performed with vectors (or matrices). More recently, large language models (LLMs) have demonstrated decent performance on bio-sequences (Ji *et al.* 2021, Dalla-Torre *et al.* 2023). However, these LLMs are susceptible to genomic variations and sequence errors; also, their memory-intensive nature hinders the efficient processing of long sequences.

To study sequences, the “gapped pattern” (or “spaced seed”) performs strikingly better than consecutive patterns, both theoretically and practically (Ilie and Ilie 2007). It tolerates substitutions, InDels, and sequence errors. The patterns have received widespread adoption in homology search, sequence alignment, and sequence assembly (Ma *et al.* 2002).

Inspired by the theory application of gapped patterns in the sequence study, here we present a sequence as a “gapped pattern graph” (GPG). The GPG has each *k*-mer as a vertex and models the relationships (or interactions) between *k*-mers as graph edges. This way, there would be no implicit ordering on the *k*-mers. A GPG is constructed as follows. Given a sequence, we create a vertex to represent each *k*-mer; the vertex is assigned a feature that encodes the frequencies of the *k*-mers within the sequence. An edge connects two vertices if their corresponding *k*-mers appear within proximity at least once in the sequence. The construction hence (1) encodes the *k*-mer distribution, and (2) enables a walk of the graph to represent a gapped pattern (in the form of a few *k*-mers with gaps in-between) within the sequence. These *k*-mers with gaps attempt to resemble the gapped patterns, enhancing the model’s capacity to characterize sequences more sensitively.

The comparison of the two GPGs gives us the similarity between the sequences they represent. Such comparisons can be made naturally through graph neural networks, which have been studied intensely in recent years. We train a Graph Convolutional Network (GCN) that transforms each GPG into a vector in a low-dimensional latent space; the vectors are then used in downstream analysis tasks. We call our resultant framework “gapped pattern-GCN” (GP-GCN).

In this study, we demonstrate the superiority of the GP-GCN framework by applying it to microbe sequences. We choose the phage sequences, i.e. virus-infecting bacteria or archaea, as the length and genomic variations of the phage sequences make their analysis challenging and representative. Under the aegis of the GP-GCN framework, we develop Graphage, a tool that performs four phage-related tasks: phage and integrative and conjugative element (ICE) discrimination, phage integration site prediction, phage lifestyle prediction, and phage host prediction. Graphage achieves state-of-the-art performance in tasks and mines distinct gapped pattern signatures for phage phenotypes. Notably, Graphage excels in scenarios where the input sequences exhibit limited homology to the training data, stemming from its inherent capacity to accommodate genomic variations. Also, we demonstrate the applicability of the GP-GCN framework by showcasing its consistent performance on sequences in tasks involving mammalian and human genomes.

## 2 Materials and methods

### 2.1 Gapped pattern graph construction

#### 2.1.1 Notation and definition

For a given sequence *S* of the alphabet {A,G,T,C} and a positive integer *k*, the *k*-mers of *S* are all the substrings of *S* of length *k*. Examples of 2-mers are *AA* and *GA*, while examples of 3-mers are *ACG* and *GAG*. There are a total of $4^{k}$ distinct *k*-mers for any positive integer *k*, and at most $\min\{4^{k},d-k+1\}$ unique *k*-mers can occur in a sequence of length *d* (d≥k). An arbitrary indexing can be imposed over the set of all *k*-mers for any fixed *k*. Such indexing can be used as indices in a vector, thus allowing the *k*-mer distribution of a sequence to be represented as a vector of integers.

#### 2.1.2 Pattern graphs

Given positive integer *k*, a “pattern graph” G(S,k) of a sequence *S* is a directed graph where each vertex corresponds to a *k*-mer of *S*. For two vertices u,v∈G(S,k), an edge from *u* to *v* is in the graph if and only if the concatenation of the two *k*-mers that the vertices correspond to, $S_{u}$ and $S_{v}$ say, exist in *S*. For example, G(ATGATGC,3) would consists of four vertices $v_{1},v_{2},v_{3},v_{4}$ corresponding respectively to the *k*-mers *ATG*, *TGA*, *GAT*, and *TGC*; it would have exactly two edges: the edges $v_{1}\to v_{1}$ and $v_{2}\to v_{4}$. Each vertex in G(S,k) is assigned a number that indicates how frequently its corresponding *k*-mer occurs in *S*; similarly, each edge is assigned a number indicating how frequently the concatenation of the two *k*-mers occurs in *S*.

Two notions of frequencies are considered for the vertex and edge features: absolute or normalized counts. An absolute count of a *k*-mer is the number of times the *k*-mer occurs in *S*; the normalized count of a *k*-mer is the number of times the *k*-mer occurs in *S* divided by the total number of *k*-mer occurrences (i.e. L−k+1). These variants of frequency are similarly defined for the edge features. We use normalized count for both vertex and edge features in this work. Notably, all conceivable vertices and edges are incorporated in the constructed graph. In cases where the corresponding *k*-mer or *k*-mer pair is absent in the sequence, it is assigned a count of zero. Supplementary Fig. S1 shows examples of this construction.

Lemma 1 Given a sequence *S* of length *L*, the pattern graph G(S,k) can be constructed in O(L) time.

#### 2.1.3 Gapped pattern graphs

To allow SNPs and InDels in a sequence, we construct a GPG as follows. Let *d* be the maximum allowed gap length. The GPG G(S,k,d) is defined in the same way as the pattern graph, except for the edges. For two vertices u,v∈G(S,k,d), an edge from *u* to *v* is in the graph if and only if the *k*-mers that the vertices correspond to, $S_{u}$ and $S_{v}$ say, exist in *S* with $S_{v}$ occurring within distance *d* after $S_{u}$. That is, $S_{u}xS_{v}$ is a substring of *S*, where *x* is an arbitrary (and possibly empty) string of length at most *d*. Each edge is assigned a vector of length d+1, the (i+1)th element of which indicates the frequency of $S_{u}yS_{v}$ in *S*, where *y* is an arbitrary string of length *i*. Varying the parameter *d* allows more flexibility in the gap length, hence increasing the expressiveness of the pattern space spanned by the GPG. Examples of this construction are given in Supplementary Fig. S2.

Just as every edge in a GPG can be considered a pattern of two *k*-mers with a gap of up to length *d* in between, every walk of length *l* on a GPG can be considered a pattern that consists of l+1 k-mers, with gaps of up to length *d* in between. Given the GPG of a sequence *S*, all subsequences of *S* can be matched to one or more gapped patterns formed by such walks. Note that the other direction of this statement is not necessarily true for patterns with more than two *k*-mers.

Lemma 2 Given a string *S* of length *L*, the GPG G(S,k,d) can be constructed in O(dL) time.

In this work, we typically let k=3 and d=2 (see Supplementary Tables S1–S4). However, for large values of *d*, we can compress the vector by letting some vector elements account for a range of gap lengths. For instance, we can let the first three elements indicate the frequencies of $S_{u}yS_{v}$ where *y* has lengths of 0, 1, and 2, respectively, but let the fourth element indicate the frequencies where *y* has lengths of 3 or 4, the fifth element indicate the frequencies where *y* has lengths from 5 to 9, and so on. Implementation details are given in Supplementary Method S1.1 and Fig. S3.

### 2.2 Neural network architecture

After constructing the GPGs from sequences, a GCN is used to transform each GPG into an embedding. We use the PyTorch Geometric implementation of GraphSAGE (Hamilton *et al.* 2017) for GCN (i.e. SAGEConv). Using the default settings, each graph convolutional layer computes the function,

(1) $$\begin{array}{c} h_{v}^{\prime }=\sigma \left(Bh_{v}+W\cdot \mathrm{mean}(\{h_{u}|u\in N(v)\})\right) \end{array}$$

where $h_{v}$ is the feature of vertex *v*, N(v) is the set of all vertices in the graph where edges incident to *v* emanate from, σ is a nonlinear activation function, mean computes the mean of the input features, and *W*, *B* are matrices to be learned. [Since GPGs are directed graphs, the use of such a function implies a spatial paradigm rather than a spectral one. Spectral GCN models for directed graphs exist (Ma *et al.* 2019, Tong *et al.* 2020, Zhang *et al.* 2021), but they are not yet generally available in standard libraries.]

As discussed in Supplementary Method S1.1, we convert edge features in the GPG into vertex features. Since the feature length of the *k*-mer vertices does not match that of the *k*-mer pair vertices in general, we use a two-step feature computation as follows. Denote a GPG as G(S,k,d)=(V,U,E), where $V=\{v_{1},v_{2},\ldots ,v_{n}\}$ is the set of *k*-mer pair vertices, $U=\{u_{1},u_{2},\ldots ,u_{n^{\prime }}\}$ is the set of *k*-mer vertices, and E={(v,u)|v∈V,u∈U}∪{(u,v)|v∈V,u∈U} is the set of edges. The input features for *k*-mer pair vertices $X_{v}=\{x_{v}|v\in V\}$ and input features for *k*-mer vertices $X_{u}=\{x_{u}|u\in U\}$ are the encoded frequencies, where $x_{v}\in R^{d+1}$ and $x_{u}\in R$. A graph convolutional layer at level *l* accepts as input a list of *k*-mer pair vertex features $H_{v}^{l}=\{h_{v_{1}}^{l},\;h_{v_{2}}^{l},\ldots ,\;h_{v_{n}}^{l}\}$ and a list of *k*-mer vertex features $H_{u}^{l}=\{h_{u_{1}}^{l},\;h_{u_{2}}^{l},\ldots ,\;h_{u_{n^{\prime }}}^{l}\}$. Clearly, $h_{v}^{0}=x_{v}$ and $h_{u}^{0}=x_{u}$. The layer outputs $H_{v}^{l+1}=\{h_{v_{1}}^{l+1},\ldots ,\;h_{v_{n}}^{l+1}\}$ and $H_{u}^{l+1}=\{h_{u_{1}}^{l+1},\ldots ,\;h_{u_{n^{\prime }}}^{l+1}\}$ which are computed as:

(2) $$\begin{array}{c} h_{u}^{l+1}=\sigma \left(B_{1}^{l}h_{u}^{l}+W_{1}^{l}\cdot \mathrm{mean}(\{h_{v}^{l}|v\in N(u)\})\right) \end{array}$$

(3) $$\begin{array}{c} h_{v}^{l+1}=\sigma \left(B_{2}^{l}h_{v}^{l}+W_{2}^{l}\cdot \mathrm{mean}(\{h_{u}^{l+1}|u\in N(v)\})\right) \end{array}$$

where $W_{1}^{l}$, $W_{2}^{l}$, $B_{1}^{l}$ and $B_{2}^{l}$ are matrices to be learned. The outputs of the graph convolutional layer at level *l* are given as inputs to the graph convolutional layer at level l+1. Note that we use $h_{u}^{l+1}$ instead of $h_{u}^{l}$ in the computation of $h_{v}^{l+1}$ (Equation 3) as a matter of preference.

The output of the final graph convolutional layer, which consists of feature vectors associated with each edge, is concatenated and given as input to a convolutional neural network (LeCun *et al.* 2015) to produce the embeddings that are used in downstream tasks.

In the present work, all downstream tasks are performed with fully connected multilayer perceptron (MLP) modeled for classification. For the phage and ICE discrimination task and the phage lifestyle prediction task, we further give the genetic features to the first layer of the MLP.

### 2.3 Training process and hyperparameters

During training, we use weighted sampling to overcome label imbalance in the training data. The sampling weight of a label is set to the inverse of its frequency in the training data. We apply mini-batch gradient descent to optimize the cross-entropy loss function:

(4) $$\begin{array}{c} L=-\frac{1}{N_{b}}\sum_{\left(x,y\right)}\sum_{c=0}^{K-1}y_{c}\;\text{log}\;p_{c}(x),\;y_{c}=\{\begin{array}{cc} 1, & \text{if}\;y=c\\ 0, & \text{otherwise}, \end{array} \end{array}$$

where $N_{b}$ is the batch size, *x* the network input, *y* the label, *K* the number of classes, and $p_{c}(x)$ the predicted probability that *x* belongs to class *c*. The Adam gradient descent algorithm is used in back-propagation with a learning rate of $10^{-4}$.

We perform a grid search (Shekar and Dagnew 2019) to tune the hyperparameters and select the settings with the best performance on validation data. The hyperparameter settings are summarized in Supplementary Tables S1–S4. The neural networks are implemented in PyTorch and PyTorch Geometric. All experiments were run on an Nvidia Tesla T4 (16G).

### 2.4 Train-validation-test split

In our experimental setup, we split the available data into distinct sets for training, validation, and testing. For the phage and ICE discrimination task, we split the NCBI (Brister *et al.* 2015) and ICEberg (Bi *et al.* 2012) datasets into training and validation sets with the ratio of 90% and 10%; we apply an independent dataset from ImmeDB as the test set. For other tasks, we split each dataset into training, validation, and test sets with proportions of 80%, 10%, and 10%. We summarize the sample distributions in each set for the classification tasks studied in this work (Supplementary Table S5). The training set is used to train the models; the validation set is applied to hyperparameter selection; the test set serves as the benchmark to evaluate the model performance.

## 3 Results

### 3.1 Overview of GP-GCN framework and Graphage

In this study, we present the GP-GCN framework to encode genome sequences. In the input preparation stage, each phage sequence *S* is converted into a GPG (see section 2.1). Each of the sequence’s constituent *k*-mers becomes a vertex and is assigned the *k*-mer’s frequency as its feature. An edge points from a vertex of the *k*-mer *s* to a vertex of the *k*-mer *t* if and only if *t* occurs after *s* at least once in *S* within a given threshold distance. Therefore, such an edge represents a pattern of the form *sxt*, where *s* and *t* are *k*-mers and *x* is an arbitrary string of length up to the given threshold. The edge is assigned the frequency of the pattern *sxt* in *S* as its feature.

**Figure 1.**
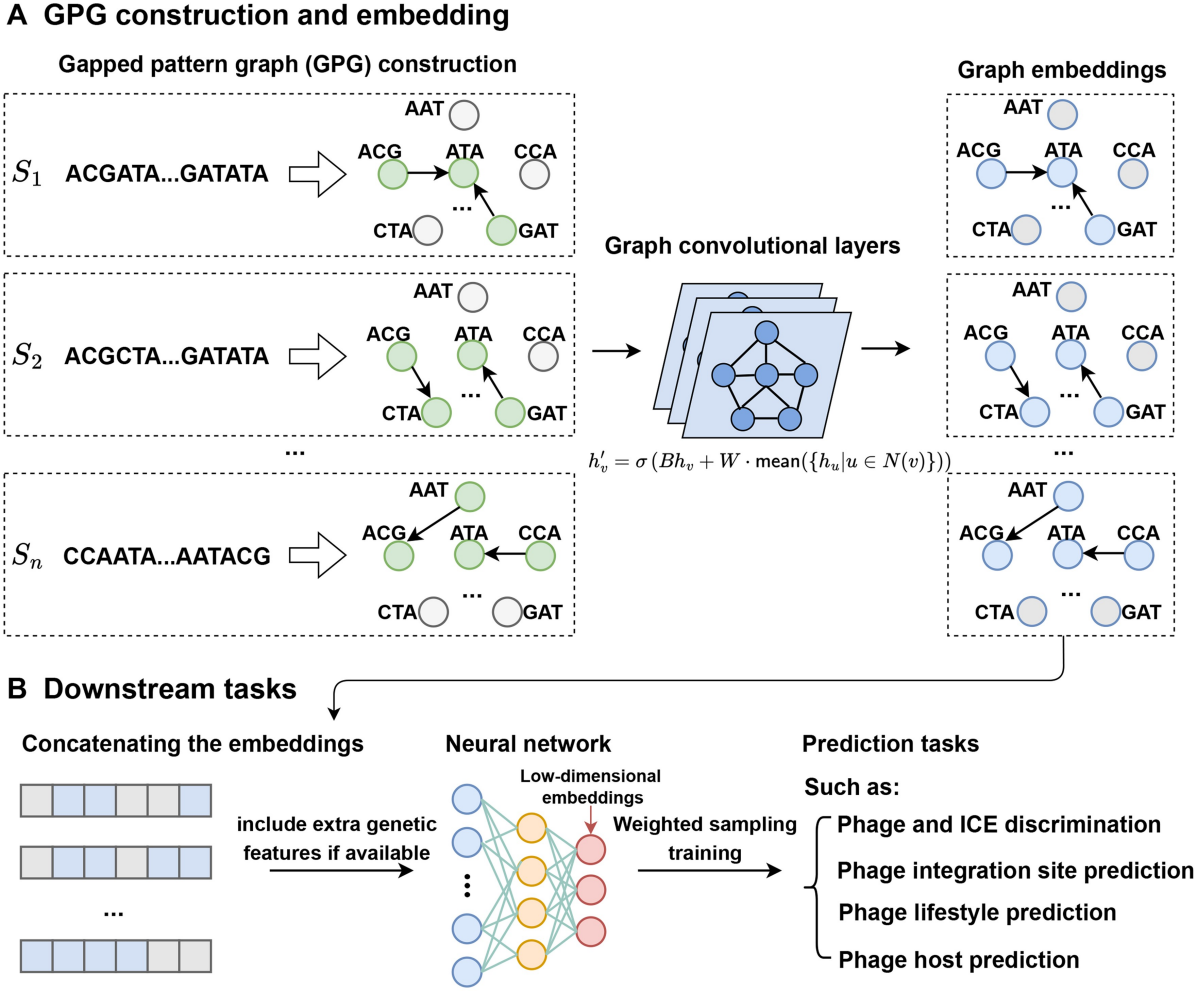
Overview of GP-GCN framework. A. A gapped pattern graph is constructed for each sequence and is embedded into latent spaces with a GCN. B. The embeddings are given as input to neural networks for downstream tasks.

The first part of GP-GCN transforms each GPG into a low-dimensional embedding (latent space vectors) using a multilayer GCN (see section 2.2) (Fig. 1A). The second part implements fully connected neural network models for prediction tasks (Fig. 1B). These models accept embeddings as input and give predictions as output for a specific task. We compare the framework with other genome encoding models, including frameworks based on LLMs (Ji *et al.* 2021, Dalla-Torre *et al.* 2023), DNA-GCN designed for DNA-protein binding site identification (Guo *et al.* 2021) and GraphLncLoc focused on long noncoding RNA (lncRNA) subcellular localization prediction (Li *et al.* 2023). We evaluate these models regarding their properties and applications (Supplementary Table S6). The GP-GCN framework features a modular design that can be flexibly applied to genomic study. The GP-GCN framework is available at https://github.com/deepomicslab/GCNFrame.

Applying the GP-GCN framework to phage sequences, we design Graphage for four phage-related tasks (Supplementary Method S1.2, Figs S4 and S5, and Table S7) and compare it with benchmark methods (Supplementary Methods S1.3 and S1.4). Five neural network models are used, each corresponding to a specific phage analysis task. The GCN and the downstream model are trained simultaneously. Graphage is available at https://github.com/deepomicslab/Graphage.

### 3.2 Phage and ICE discrimination

We use the sequences from NCBI (Brister *et al.* 2015) and ICEberg (Bi *et al.* 2012) as the training set for phage and ICE discrimination. A classifier is used to distinguish between the phage and ICE sequences given their respective GP-GCN embeddings. Following the practice introduced in GPD (Camarillo-Guerrero *et al.* 2021), we include information on gene density and hypothetical protein fraction as input to the classifier in addition to embeddings. We optimize two hyperparameters by grid search: (1) the maximum allowed length *d* for the gap between *k*-mers, and (2) the number of layers *l* in the GCN. The results showed that d=1 and l=3 achieved the best performance (Supplementary Fig. S6). More hyperparameter settings can be found in Supplementary Table S1.

We first compared the effects of using the GP-GCN embeddings with those of using other input types. When tested in the ImmeDB database (Jiang *et al.* 2019), Graphage outperformed AF-based, word2vec-based, and sequence-based models, with a 2.31% increase in average accuracy, 4.93% increase in F1-score, and 0.32% increase in average area under ROC, compared to the respective second-highest scores (Supplementary Table S8). The sequence-based model gave the worst performance, consistent with our argument that the method is unsuitable for sequences of variable lengths.

Since a GPG contains information on frequencies of both the *k*-mers (vertex feature) and the gapped patterns of two *k*-mers (edge feature), it is worth questioning if the latter could be solely responsible for the performance of Graphage. To answer this question, we performed tests with the frequencies of the gapped patterns as input to several conventional machine learning models (Supplementary Method S1.3). The results show that this is not the case (Supplementary Table S8).

We then compared Graphage to the model introduced in GPD (Camarillo-Guerrero *et al.* 2021) for phage and ICE discrimination. We found that the GPD model is prone to misclassify ICEs as phages, leading to lower accuracy and F1 scores.

Finally, there is the concern of whether Graphage’s classifier depended more on the embeddings or the genetic features (i.e. gene density and hypothetical protein fraction) for its output. To evaluate this possibility, we re-evaluated Graphage with only the embeddings or only the genetic features as input. We found that the best performance is achieved with both inputs, showing that they jointly contributed to the performance (Supplementary Fig. S7).

### 3.3 Phage integration site prediction

Phage integration site inference is complicated by the fact that circular phage genomes can integrate into the bacterial genome as prophages. To distinguish them, we trained two models that predict phage integration sites on phage genomes and bacterial genomes. A dataset is prepared for each model. The dataset for phage integration sites consists of 21 117 positive and 21 117 negative samples; that for bacterial integration sites consists of 17 550 positive and 17 550 negative samples. We used 90% of the data as training and validation sets. In addition to the allowed gap length *d* and the number of graph convolutional layers *l*, we also optimized the window length *w* (i.e. the input sequence length). The results show that d=2, l=3, and w=600 gave the best performance for integration site prediction for both models (Supplementary Figs S8 and S9). More hyperparameter settings are shown in Supplementary Table S2.

When evaluated with the respective testing set, both models obtained prediction precisions >0.8, significantly outperforming the models based on AF, word2vec, sequence, LLMs, or gapped pattern frequencies under various metrics (Supplementary Tables S9 and S10). For integration site prediction in phage sequence, Graphage has a 2.70% lead on average accuracy over the runner-up’s score; this lead is 2.50% with bacteria genomes. We observe that the sequence-based model performed worse when inputs are of variable length than when they are of the same length, demonstrating the disability of the representation in handling length variations.

### 3.4 Phage lifestyle prediction

In lifestyle prediction, we want to classify phages by whether they are virulent or temperate. Since sequences alone are likely insufficient for this classification, we supplemented the classifier with additional information by using a dataset that contains 206 lysogeny-associated proteins as established by BACPHLIP (Hockenberry and Wilke 2021). We used HMMER (Johnson *et al.* 2010) to check the presence of the 206 proteins in each sequence and encoded the information into a 206-dimensional vector, with 0 for absence and 1 for presence. The maximum allowed gap length (*d*) and the number of graph convolutional layers (*l*) were optimized through grid search. The model with d=2 and l=4 achieved the best classification performance (Supplementary Fig. S10). More hyperparameters can be found in Supplementary Table S3.

Graphage gave the highest average performance among all benchmark methods, including AF-based, word2vec-based, sequence-based, and gapped pattern frequencies-based models, as well as two phage lifestyle prediction tools, BACPHLIP (Hockenberry and Wilke 2021) and DeePhage (Wu *et al.* 2021) (Supplementary Table S11). Compared to the other methods, Graphage demonstrated large improvements in accuracy and F1-score metrics.

To determine the contribution of the additional information, we trained two Graphage models, one with only embeddings and one with only information on lysogeny-associated proteins. Although these models performed worse than when both information were available (Supplementary Fig. S11), the model with only protein information had an edge over that with only embeddings.

### 3.5 Phage host prediction

Phage host prediction is a multiclass prediction problem. Our constructed dataset consists of 107 classes, each for a host species. We use 90% of the data for training and validation. Accuracy, weighted F1-score, and macro F1-score were used as evaluation metrics (Supplementary Method S1.4). Under these metrics, the model with d=2 and l=1 achieved the best performance (Supplementary Fig. S12). More hyperparameter settings are shown in Supplementary Table S4.

In addition to AF-based, word2vec-based, sequence-based, and gapped pattern frequencies-based models, we also evaluated the performance of four available tools, namely HostPhinder (Villarroel *et al.* 2016), VirHostMatcher (Galiez *et al.* 2017), WIsH (Galiez *et al.* 2017), and DeepHost (Wang *et al.* 2022). In the tests using VirHostMatcher and WIsH, the host reference database consists of all 107 bacteria genomes from NCBI; the bacteria with a sequence most similar to that of the phage sequence is taken as the prediction.

Graphage outperformed all the methods compared (Supplementary Table S12). Only the AF-based model achieved performance comparable to Graphage in both accuracy and weighted F1 score; this could be due to the *k*-nearest neighbors method used in the model, which generalizes well to multiclass prediction tasks. All the methods other than VirHostMatcher scored much worse in macro F1-score than in weighted F1-score, since predicting the samples from rare classes is a much more challenging task.

### 3.6 Necessity of GCN component

To verify the necessity of the GCN component within the GP-GCN framework, we performed an ablation study by removing the GCN while keeping other components the same. The inputs of the ablation models are also GPGs. Specifically, the edge features, i.e. the gapped pattern frequencies, are concatenated and fed into a fully connected neural network for downstream prediction tasks (Supplementary Fig. S13). We trained new models for the four tasks and compared the performance with Graphage. The results in Supplementary Table S13 show that the models with GCN achieved superior performance on all four tasks.

**Figure 2.**
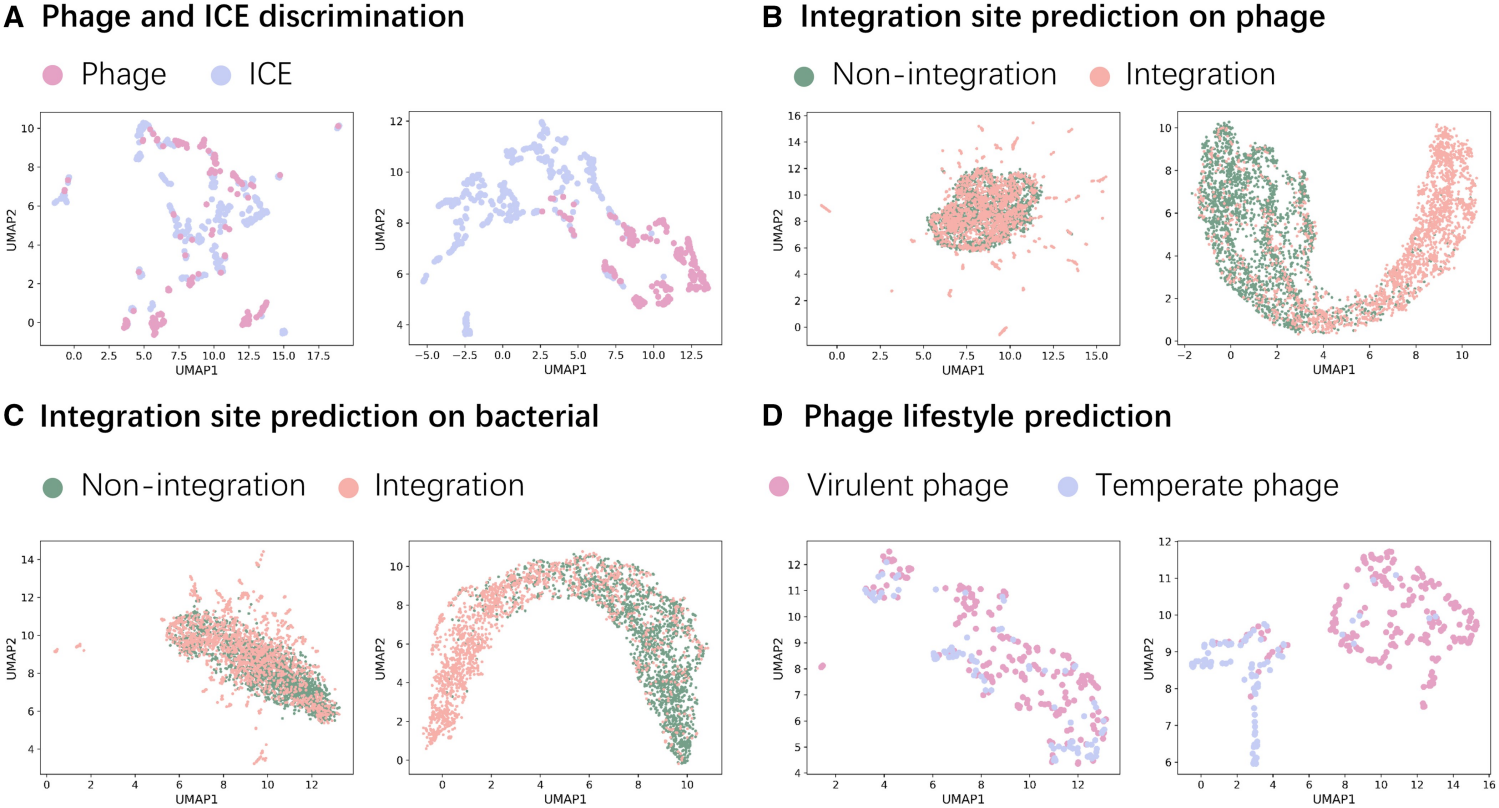
UMAP projections of the output from models with GCN and without GCN. For all subfigures A, B, C, and D, the latent features output by the models (prior to the final layer) without GCN are shown on the left, while that output by the models with GCN is shown on the right. Better separation between classes can be observed in the output of the models with GCN.

We visually inspect the influence of GCN on the final output of Graphage on a binary classification task. More precisely, we examine the outputs of Graphage right before its final, fully connected layers, to see how well the outputs of sequences from the same class form clusters; we compare the clusters for the case when GCN is used and when it is not. To enable visualization, we use uniform manifold approximation and projection (UMAP) (McInnes *et al.* 2018) to project the output values for each sequence into two dimensions. As shown in Fig. 2, the outputs when GCN is used form more clearly defined clusters in accordance with their labels.

Next, we explore the implications of performance improvement provided by the GP-GCN framework. The improvement is mainly attributed to the model’s ability to deal with the sequences with limited homology to the training data (Supplementary Result S2.1 and Fig. S14). By conducting the ablation study, we demonstrate that the model’s performance on novel sequences decreases significantly upon removal of the GCN component; also, the incorporation of gapped patterns leads to more accurate prediction on novel sequences (Supplementary Table S14).

### 3.7 Application of GP-GCN framework on sequences beyond phage genomes

To illustrate the applicability of the GP-GCN framework, we assess the robustness of GCN-produced embeddings (Supplementary Method S1.5) and the scalability of the GP-GCN framework. The findings highlight the advantages of our GP-GCN framework in handling sequence variations and processing large datasets effectively (Supplementary Results S2.2 and S2.3 and Fig. S15).

Additionally, we apply the framework with default hyperparameters (Supplementary Table S15) on three tasks that involve mammalian and human genomes, including species classification with major histocompatibility complex (MHC), regulatory item identification, and lncRNA localization prediction (Supplementary Fig. S16 and Table S16). Our framework outperforms the benchmark methods across the tasks, demonstrating its reliable performance on multi-species genomes (Supplementary Result S2.4 and Tables S17–S19). Also, we showcase the robustness of our framework across sequences with variable lengths (Supplementary Result S2.5 and Fig. S17). These results further underscore the superiority and applicability of our GP-GCN framework.

### 3.8 Gapped pattern signature and regulatory motif mining

From the models of Graphage, we calculate the contribution scores for the patterns and pattern groups (Supplementary Method S1.6 and Fig. S18) to mine informative pattern signatures for the phage-related tasks (Supplementary Result S2.6 and Figs S19 and S20).

**Figure 3.**
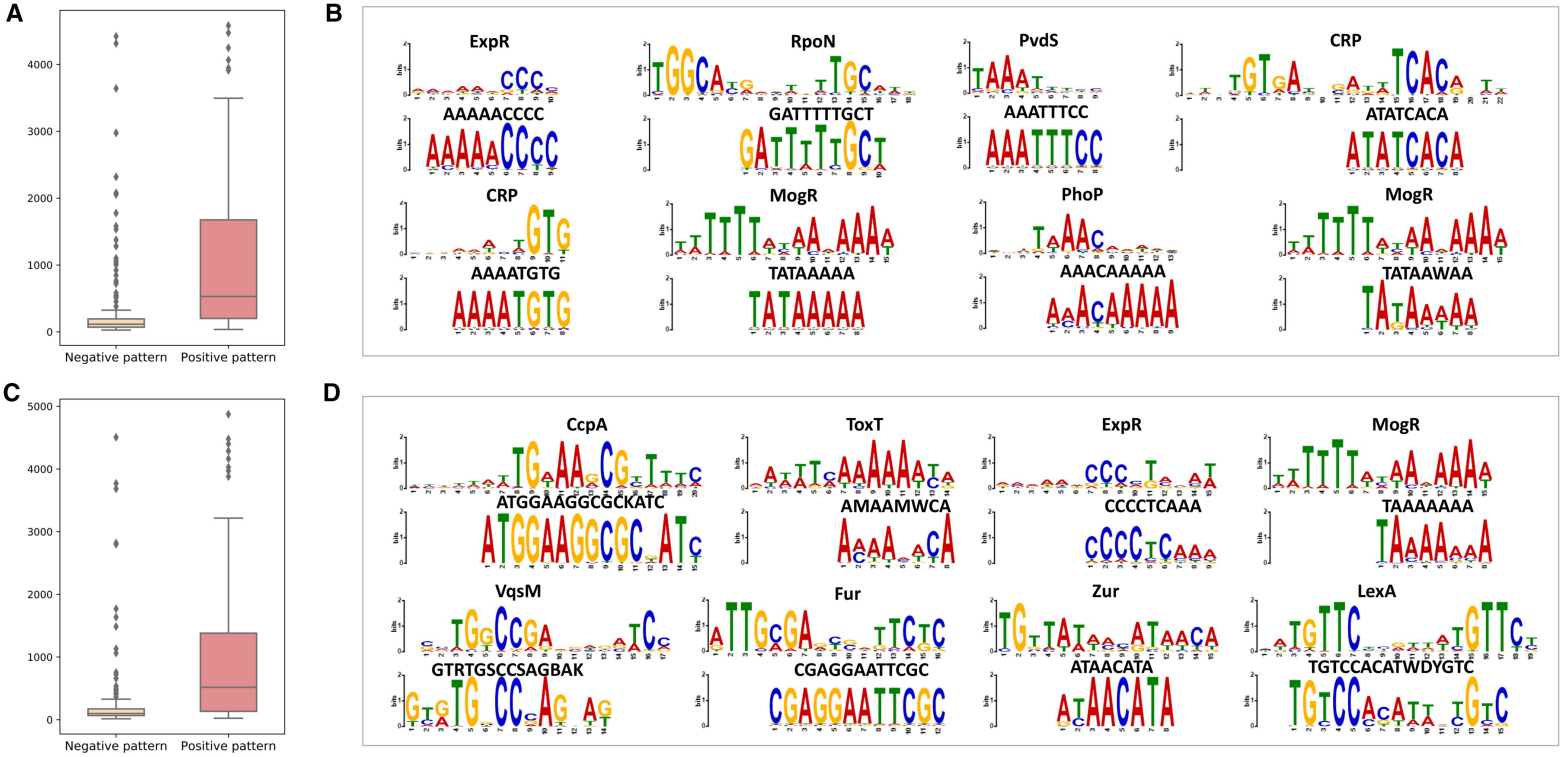
Motifs discovered in the integration site dataset match the TF-binding profiles in CollecTF. In the boxplots, we show the number of positive sequences for the enriched motifs with lower contribution scores than the baselines and higher contribution scores than the baselines in phage integration sites (A) and bacterial integration sites (C). For the motifs with higher contribution scores than baselines in phage integration sites (B) and bacterial integration site (D), we show the sequence logo alignment between the motif in CollecTF (top) and the motif in the integration site (bottom) for the top eight motifs according to the alignment *P*-value.

Additionally, we investigated the regulatory motifs to study the phage integration mechanism. First, we applied STREME (Bailey 2021) to discover the motifs enriched in the sequences of the integration site with negative sequences as control. For each motif, we calculated the contribution score by removing all the possible gapped patterns in the motif from the sequences and calculating the mean absolute error between the two output probabilities. We also calculated a baseline score for each motif by removing the same number of randomly chosen gapped patterns. Then, we identified the informative motifs that had higher contribution scores than their baselines. As a result, we obtained 57 informative motifs from phage integration sites and 51 motifs from bacterial integration sites. These informative motifs match with more positive sequences than the motifs with lower contribution scores than baseline (Fig. 3A and C).

Next, we utilized Tomtom (Gupta *et al.* 2007) to compare these motifs against the CollecTF dataset (Kılıç *et al.* 2014), which collects the transcription factor (TF)-binding sites in Bacteria. We present the sequence logo alignment for the eight motifs with the lowest alignment *P*-value (Fig. 3B and D). The identified TFs are known to be involved in viral activities such as replication and integration (Supplementary Result S2.7).

## 4 Discussion

The bio-sequences are known for their frequent genetic variations, which complicates any analysis that requires a homology search. Current computations of homology are mainly alignment- or AF-based. Alignment-based methods face runtime complexity issues, while AF-based methods compromise important information in the sequences. As bio-sequence databases become large and sequence diversity increases, such compromises are beginning to limit genomic analyses.

In this work, we propose a novel method for succinct biosequence representation, where each sequence is represented as a *k*-mer-encoding graph called GPG. To enable efficient comparison of these GPGs we exploit recent advances in graph neural networks to filter such graphs into low-dimensional embeddings. These ideas give rise to a framework that we call GP-GCN. In the construction of GPGs, we incorporate each conceivable vertex and edge to ensure that the output of the final graph convolutional layer can be concatenated to the same dimensionality for input into the subsequent network. Another common alternative approach involves including solely the vertices and edges that appear in the sequence. In such a case, each GPG may have a distinct subset of nodes, necessitating the use of a pooling function to average over the edge embeddings for a uniform output dimension across samples. Our experiment results showcase that our GP-GCN framework outperforms the strategy of selectively including the vertices and edges that appear in the sequence, particularly in challenging tasks such as host prediction (Supplementary Fig. S21).

In our experiments, the models based on the GP-GCN framework consistently outperform those based on other encoding methods across various tasks. Our ablation studies elucidate that the performance improvement sources from the GCN component enable the model to be generalized to novel sequences. Different from conventional encoding methods, which typically encode genomes into vectors or matrices, the GP-GCN framework encodes genomes into GPGs, which preserves information pertaining to gapped patterns within the genome. The framework further leverages graph convolutional layers to model interactions between these gapped patterns, thus enhancing its adaptability to diverse genomic contexts and variations.

The application of representation learning to biological sequences is not new. For instance, word2vec, a widely used natural language processing technique, has been applied to obtain embeddings from sequences of the human genome, as well as solve problems such as species identification, methyladenosine site prediction, and MHC-binding site prediction. However, word2vec is based on encoding local contextual information of sequence segments and has no provision for representing global information of entire sequences. In our tests, the GP-GCN framework consistently outperformed word2vec-based models.

We expect the GP-GCN framework to be useful beyond the demonstrated prediction tasks, due to its many desirable properties, such as robustness with respect to genomic variations and high computational efficiency. Our immediate future plan is to attempt the framework on more types of sequences, e.g. RNA and protein sequences, as well as on more complex downstream tasks, such as sequence generation or sequence restoration. One application, clustering of genome sequences, is of fundamental importance to many studies such as phylogenetics, and has spurred the development of many tools. The ability to cluster biosequences more accurately, and on larger scales, would benefit many researchers.

Other possibilities abound for the practical use of the GP-GCN framework. For instance, trained GP-GCN models encode important domain knowledge of the tasks that they were trained for. Mining gapped patterns from the models trained in the four tasks in Graphage has allowed us to uncover patterns that are possibly relevant to phage lifestyle. Another possible use of the framework is in the discovery of partial similarities between GPGs. This may be useful in situations where the GPGs are constructed from genetic materials consisting of multiple species, such as in metagenomic studies. In such a case, partial GPG similarity would suggest a similar subcomposition of the two inputs.

## Supplementary Material

btae188_Supplementary_Data

## Data Availability

The data underlying this article will be shared on reasonable request to the corresponding author.
