# Correction to: Coding genomes with gapped pattern graph convolutional network

**DOI:** 10.1093/bioinformatics/btaf336

**Published:** 2025-06-22

This is a correction to: Ruo Han Wang, Yen Kaow Ng, Xianglilan Zhang, Jianping Wang, Shuai Cheng Li, Coding genomes with gapped pattern graph convolutional network, *Bioinformatics*, Volume 40, Issue 4, April 2024, https://doi.org/10.1093/bioinformatics/btae188

In the originally published online version of this manuscript, the funding project number was erroneous. This should read: “JCYJ20220818101201004” instead of: “20220814183301001”.

This error has been outlined only in this correction notice to preserve the version of record.

