# Supplementary material for: Coding genomes with gapped pattern graph convolutional network
Source: Bioinformatics. 2024 Apr 11;40(4):btae188. doi: 10.1093/bioinformatics/btae188 (PMC11034989; doi:10.1093/bioinformatics/btae188)
Supplement: btae188_Supplementary_Data [file btae188_supplementary_data.pdf]

Coding genomes with gapped pattern graph convolutional network

Ruo Han Wang, Yen Kaow NG, Xianglilan Zhang, Jianping Wang, and Shuai Cheng Li

April 3, 2024

Contents

S1 Supplementary methods **3**
S1.1 Implementation details . . . 3
S1.2 Benchmark datasets . . . 3
S1.3 Benchmark methods . . . 3
S1.4 Performance evaluation metrics . . . 4
S1.5 Simulating datasets with genetic variations . . . 5
S1.6 Contribution score of gapped patterns and motifs . . . 5

S2 Supplementary results **6**
S2.1 Improved performance on novel sequences . . . 6
S2.2 Robustness of GCN-produced embeddings . . . 6
S2.3 Scalability of the GP-GCN framework . . . 6
S2.4 Application of the GP-GCN framework on other tasks . . . 6
S2.4.1 Species classification with MHC . . . 6
S2.4.2 Regulatory item identification . . . 7
S2.4.3 lncRNA localization prediction . . . 7
S2.5 Performance of GP-GCN framework on sequences with various lengths . . . 7
S2.6 Mining gapped patterns of more significant influence in prediction tasks . . . 8
S2.7 Transcription factor associations at phage and bacterial integration sites . . . 8

S3 Supplementary figures **9**

S4 Supplementary tables **24**

List of Figures

S1 Examples of pattern graph construction. . . . 9
S2 Examples of gapped pattern graph construction. . . . 10
S3 An example of bipartite pattern graph construction. . . . 11
S4 Four benchmark datasets are prepared, each for a phage-related task. . . . 12
S5 The length distributions of the input sequences for the phage-related tasks. . . . 12
S6 The effect of hyperparameters on the performance of phage and ICE discrimination. . . . 13
S7 The effect of input features on the performance of phage and ICE discrimination. . . . 13
S8 The effect of hyperparameters on the performance of integration site prediction on phage genomes. . . . 14
S9 The effect of hyperparameters on the performance of integration site prediction on bacteria genomes. . . . 14
S10 The effect of hyperparameters on the performance of phage lifestyle prediction. . . . 15
S11 The effect of input features on the performance of lifestyle prediction. . . . 15
S12 The effect of hyperparameters on the performance of phage host prediction. . . . 16

S13 Illustration of the ablation model. 17
S14 Analysis of the sequences that could be correctly classified by Graphage but not by the MLP model. 18
S15 The distance between the vectors of the original sequences and mutated sequences from different embedding. 18
S16 The length distributions of the input sequences for the three extended applications. 19
S17 Model performance on sequences with variable lengths. 19
S18 Contribution score calculation. 20
S19 The contribution score analysis of the gapped patterns for phage lifestyle prediction. . . . 21
S20 The contribution score analysis of the gapped patterns for phage and ICE discrimination, phage integration site prediction, and bacterial integration site prediction. 22
S21 Performance comparison of the models from the original GP-GCN framework and the models when edge embeddings from the final graph convolutional layer are average pooled. 23

List of Tables

S1 The hyperparameters used for phage and ICE discrimination in Graphage. 24
S2 The hyperparameters used for integration site prediction in Graphage. 24
S3 The hyperparameters used for phage lifestyle prediction in Graphage. 25
S4 The hyperparameters used for phage host prediction in Graphage. 25
S5 The number of samples in training, validation, and test sets for each task. 26
S6 Feature and application comparisons for the genome encoding models.*Nucleotide Transformer and DNABERT accept DNA sequences with maximum lengths of 512 *bp* and 6 *kb*, respectively. 26
S7 Summary of benchmark datasets in this study. 27
S8 Comparing Graphage with other models/tools on phage and ICE discrimination tasks. . . 27
S9 Comparing our graph model with other models/tools on phage integration site prediction tasks. 28
S10 Comparing our graph model with other models and tools on bacterial integration site prediction tasks. 28
S11 Comparing our graph model with other models and tools on phage lifestyle prediction tasks. 29
S12 Comparing our graph model with other models and tools on phage host species prediction tasks. 29
S13 The performance comparison of models with and without graph convolutional layers. . . . 30
S14 For the ablation study, we first align the test sequences to the training set with BLAST (e-value $< 10^{-5}$). Among the 606 test sequences, 253 sequences failed to align with the training set in this case, which are regarded as novel test sequences. Then we evaluate the performance of three models, the complete GP-GCN framework, the framework without the GCN module, and the framework with d={0}, on overall test sequences and novel test sequences. 30
S15 The default hyperparameters of the GP-GCN framework. 30
S16 The statistics of the three extended applications for the GP-GCN framework. 31
S17 Comparing our graph model with other models on species classification with MHC task. . 31
S18 Comparing our graph model with other models on regulatory item identification task. . . 31
S19 Comparing our graph model with other models on lncRNA localization prediction task. . 32

S1 Supplementary methods

S1.1 Implementation details

GP-GCN framework uses GCN to transform each GPG into an embedding. However, current GCN software libraries treat edges and vertices differently, whereas our formulation prefers that they be treated equally. In particular, we want edge features to be modified in the same way as vertex features.

To overcome this limitation, we insert a vertex on every edge in the GPG and assign the feature of the edge to the new vertex. The new vertex maintains the connections of the original edge; hence every edge becomes a new vertex of in-degree one and out-degree one. We call these new vertices *k-mer pair vertices* to distinguish them from the *k-mer vertices* in the original GPG. This conversion does not change the information flow of the original GPG; on the other hand, all the features on the GPG are now associated with vertices, thus allowing their manipulation using standard GCN libraries.

From the construction, each k-mer vertex is only connected to k-mer pair vertices, and vice versa. An edge is set to point from a k-mer vertex to a k-mer pair vertex, then back to a k-mer vertex. An example of this construction is given in Supplementary Figure S3.

S1.2 Benchmark datasets

We collected seven published datasets for the four phage-related tasks (Supplementary Figure S4 and Table S7). For the task of phage and ICE discrimination, we used the phage genomes from NCBI [1] and ICE [2] sequences from ICEBerg as the training set, following the data preparation process of GPD [3]. An independent dataset, ImmeDB [4], including ICE and prophage sequences, was used as the test set. We applied Prokka [5] for sequence annotation to obtain information on gene density and hypothetical protein fraction. For the task of phage integration site prediction, we used a temperate phage dataset with precise boundary information [6]. We extracted the sequences of integration sites from both phage and bacterial genomes to use as positive sequences, and randomly chose sequences of the same length that are 1 *kb* away from any integration site on the phage and bacteria genomes for negative sequences. For phage lifestyle prediction, we downloaded phage sequences with empirical lifestyle data [7] and tagged them with the lifestyle-related protein domains selected by BACPHLIP [8]. We applied Glimmer [9] to identify the genes in phage genomes and HMMER [10] to search for the protein domains. For phage host prediction, we combined one dataset mined from metagenomic data [3] and one dataset mined from bacterial data [6] with their host information. We removed the phages with unidentified hosts and kept only the taxonomies with more than 20 phage genomes. All redundant sequences were removed from each of the seven datasets.

S1.3 Benchmark methods

A GPG contains information of both the frequencies of k-mers (vertex feature) and gapped patterns of two k-mers (edge feature). The former corresponds to the traditional AF-based input (i.e., k-mer distribution), while the latter is unique to GPG. To show that the combination of these information (in the form of the GPG) is beneficial to the prediction tasks studied in this work, we compare Graphage to similar models that accept respectively (1) AF-based input, (2) word2vec-based input, (3) sequence-based input, and (4) distribution of all the gapped patterns of k-mer pairs. We want these inputs to provide at least the same amount of information available in the GPGs. Since we use GPGs of 3-mers, we use 6-mers distribution as the AF-based input, and we use 6-mers for both the word2vec- and sequence-based input. We use 3-mers for the gapped patterns of k-mer pairs. Below are the implementation details of the models for each of these inputs.

- The AF-based models are written using alfpy [11]. We represent each sequence with a 6-mer frequency vector, then calculate the distances between the sequences in the training set and the sequences in the test set. Prediction for a test sequence is performed by returning the label of its nearest neighbor in the training set.
- For word2vec, we apply Gensim [12] to learn the 6-mer vector representations with the CBOW algorithm from the training sequences. Then a pooling strategy over the 6-mers is applied, and an

optimized fully-connected neural network is used for the final prediction.

- For sequence-based methods, every sequence is converted into a vector of a fixed length. More precisely, each sequence is straightforwardly transformed into a $4 \times L$ matrix with one-hot encoding (A is encoded as $(1,0,0,0)$; C as $(0,1,0,0)$; G as $(0,0,1,0)$; T as $(0,0,0,1)$). For phage and ICE discrimination, phage lifestyle prediction, and phage host prediction, we set L to 50,000, which is the median length of phage sequences. Sequences shorter than 50 *kb* are padded with trailing zeros, while sequences longer than 50 *kb* are discarded. An optimized fully-connected neural network is used for the final prediction.
- For large language models, we include Nucleotide Transformer [13] and DNABERT [14] as benchmark methods. For pre-trained models, we download the 500M multi-species pre-trained model for Nucleotide Transformer and DNABERT6 for DNABERT. Subsequently, we fine-tune these models with the training data for each task. The training procedures adhere to established tutorials, and the models with the optimal performance on validation data are selected for comparison. Nucleotide Transformer accepts inputs with a maximum length of 6 *kb*, so we include them in the phage integration site prediction task and species classification with MHC task; DNABERT accepts inputs with a maximum length of 512 *bp*, so we include them in the phage integration site prediction task with a window length of 500 *bp*.
- Since the gapped pattern graph of k-mer pairs is a new form of input, we evaluated several conventional machine learning models in order to identify the ones that are most suitable for it. The models examined are (1) SVM (with both linear function and radial basis function kernel), (2) k-nearest neighbors, (3) logistic regression, (4) AdaBoost, (5) decision tree, (6) random forest, and (7) MLP. We found that the best performances were achieved with k-nearest neighbors, random forest, and MLP; hence we only show the results with these three models.

We furthermore compared Graphage to other state-of-the-art tools as follows:

- GPD [3] was used for comparison in phage and ICE discrimination.
- (Phage integration site prediction is a novel task with no currently available tools.)
- BACPHLIP [8] and DeePhage [15] were compared in phage lifestyle prediction.
- HostPhinder [16], VirHostMatcher [17], WIsH [17] and DeepHost [18] were compared in phage host prediction.

S1.4 Performance evaluation metrics

The classification performances for all four tasks considered in this work are evaluated through accuracy, F1-score, and receiver operating characteristic (ROC) curve.

The F1-score is defined as

$$\text{F1-score} = \left(\frac{\text{Recall}^{-1} + \text{Precision}^{-1}}{2}\right)^{-1} \tag{S1}$$

The metric is inapplicable for phage host prediction, which is a multi-class classification task. In this case, we measure weighted F1-score and macro F1-score instead.

$$\text{weighted F1-score} = \sum_{i=1}^{K} \frac{N_i}{N} \text{F1-score}_i \tag{S2}$$

$$\text{macro F1-score} = \frac{1}{K} \sum_{i=1}^{K} \text{F1-score}_i \tag{S3}$$

where K is the number of classes, N_i the number of samples in class i, and N is the total number of samples.

S1.5 Simulating datasets with genetic variations

We simulated four kinds of genetic variations to evaluate the robustness of GCN-produced embeddings.

- Insertion: We randomly generated a DNA sequence from the deoxyribonucleotides dictionary {A, C, G, T} with length ranging from one to the maximum variation length. Then we randomly chose a site on the original sequence to insert the generated sequence.
- Deletion: We randomly chose a sequence ranging from one to the maximum variation length on the original sequence and removed it.
- Inversion: We randomly chose a sequence ranging from one to the maximum variation length on the original sequence and replaced it with the reverse complementary sequence.
- Translocation: We randomly chose a sequence ranging from one to the maximum variation length on the original sequence and moved it to another randomly chosen site on the original sequence.

We simulated three datasets with different variation rates and lengths. Each sequence in the first dataset contains eight variations, including two insertions, two deletions, two inversions, and two translocations. The variation lengths vary from 1 bp to 10 bp. Each sequence in the second dataset contains 20 variations, including five insertions, five deletions, five inversions, and five translocations. The variation lengths vary from 1 bp to 50 bp. Each sequence in the third dataset contains 40 variations, including ten insertions, ten deletions, ten inversions, and ten translocations. The variation lengths vary from 1 bp to 100 bp.

We collected one hundred phage sequences from NCBI as the original sequence; ten sequences were simulated with variations for each original sequence in each dataset. Thus, each dataset contains one thousand sequences with variations.

S1.6 Contribution score of gapped patterns and motifs

As mentioned, every edge of a GPG represents a gapped k-mer pair pattern. Some of these gapped patterns may exert more influence in a prediction task than others. To investigate this possibility, for a given input sequence, we remove the feature of an edge from the sequence's GPG and examine how that influences the prediction accuracy of a trained model on the sequence. More precisely, given a phage sequence S, we first obtain the trained output probability for the class that S belongs to. Then, we obtain the GPG of S and set the feature of a given edge in the GPG to zero, then obtain the new corresponding output probability; the mean absolute error (MAE) between the two probabilities provides us with a contribution score for the gapped pattern that corresponds to the given edge, indicating its influence on the prediction. This is repeated for every sequence and every edge of the GPG of the sequence. The total contribution of each gapped pattern is aggregated from all the differences resulting from its edge being set to zero (Supplementary Figure S18 A).

However, similar patterns are likely to influence the model simultaneously; thus, some patterns may have low contribution scores even with great influence on the model prediction. To remove this bias, we first sort the patterns in descending order according to their contribution scores. With this order, we select representative patterns, requiring a representative pattern to have Hamming distance ≥ 2 from other representative patterns. In this way, we select a distinct pattern with a high contribution score to represent a group. Next, we assign the remaining pattern to the group with a similar representative pattern (Hamming distance ≤ 1). We annotate each group with the contribution score of the representative pattern (Supplementary Figure S18 B).

For a given motif, we can also obtain the contribution score by removing all the possible gapped patterns in the motif from the sequences and calculating the MAE between the two output probabilities (Supplementary Figure S18 C). The pattern and motif scoring functions are included in the GP-GCN package.

S2 Supplementary results

S2.1 Improved performance on novel sequences

For the phage and ICE discrimination task, we collect 33 sequences that are correctly identified with Graphage but misclassified with the MLP model, which demonstrates the second-best performance. We align these sequences to the training set with BLAST (e-value $< 10^{-5}$). 18.18% of the sequences can be aligned to the training set in this case, whereas 62.29% of the sequences that can be correctly classified with both Graphage and MLP are aligned to the training set (Supplementary Figure S14 A). Additionally, the sequences only correctly classified by Graphage exhibit shorter alignment lengths (Supplementary Figure S14 B). The results indicate that the GP-GCN framework is able to deal with the novel sequences that have less similarities with the training set.

S2.2 Robustness of GCN-produced embeddings

We evaluated the robustness of GCN-produced embeddings through large-scale simulated sequences. Two criteria were used to evaluate the embeddings: (1) similar sequences should have similar embeddings and vice versa, and (2) embeddings should remain the same under a reasonable amount of genomic variations. We randomly selected 100 phage sequences from NCBI and simulated their genomic variants using insertion, deletion, inversion, and translocation. We generated three datasets with different variation rates and lengths (See Supplementary Method S1.5 for construction details of the simulated dataset).

To examine the criteria, we obtained embeddings using the GP-GCN component of the model trained for the phage and ICE discrimination task. The dissimilarity between two embeddings is computed as the Euclidean distance between the embeddings. As shown in Supplementary Figure S15, distances between the embeddings are small, even with large variations in the simulated sequences. We compared these distances to the case where the GP-GCN is replaced with a GCN without a gap and a CNN to embed the sequences into vectors of the same dimension as the GP-GCN-produced embeddings. For all three datasets, the GP-GCN framework gave embeddings with smaller distances between the original and the mutated sequences.

S2.3 Scalability of the GP-GCN framework

Due to the very large number of biological sequences in phage analysis, any method for analyzing phage sequences must be capable of handling millions of sequences. The GP-GCN encoding framework is learning-based; after the initial training, the model can perform predictions in runtime linear to the number of sequences to infer. Additionally, each gapped pattern graph can be constructed in time polynomial to the sequence length. The computational efficiency makes the framework suitable for handling large datasets.

S2.4 Application of the GP-GCN framework on other tasks

To demonstrate the extensive applicability of the GP-GCN framework, we further evaluate the framework with default hyperparameters on three tasks that involve sequences beyond phage genomes. The tasks include species classification with major histocompatibility complex (MHC), regulatory item identification, and long non-coding RNA (lncRNA) localization prediction (Supplementary Figure S16 and Table S16).

S2.4.1 Species classification with MHC

The major histocompatibility complex (MHC) genes encode proteins that play critical roles in the immune systems of various animals. These genes are highly polymorphic [19] and species-specific [20]. Polymorphism within MHC genes arises from various mechanisms such as gene duplication, recombination, and balancing selection, resulting in an extensive repertoire of allelic variants within populations [21]. Therefore, the MHC genes can serve as markers to identify species and study the diversity of populations.

We collect 8,576 MHC sequences of nine species from the IPD-MHC database [20], including 3,041 sequences from Macaca fascicularis, 2,338 sequences from Macaca mulatta, 829 sequences from Macaca

nemestrina, 738 sequences from Bos sp., 495 sequences from Sus scrofa, 357 sequences from Pan troglodytes, 296 sequences from Papio anubis, 247 sequences from Ovis aries, and 235 sequences from Callithrix jacchus. Then we apply the GP-GCN framework to classify the species from the MHC sequences.

With 10% of the data set aside as test data, the GP-GCN framework achieves an accuracy of 0.687 for the nine-classes classification task, increasing the performance of benchmark models by 2.4-23.2%. Furthermore, the evaluations also reveal significant improvements in weighted and macro F1-score (Supplementary Table S17).

S2.4.2 Regulatory item identification

Identifying regulatory elements in the human genome, such as enhancers and promoters, is fundamental to studying gene expression and regulation. Regulatory elements play crucial roles in modulating gene transcription by interacting with transcription factors and other regulatory proteins. Enhancers facilitate gene expression from a distance, whereas promoters are regions where RNA polymerase binds to initiate transcription [22]. Traditional approaches for regulatory item identification rely on experimental techniques, which are often time-consuming, labor-intensive, and with limited capacity to capture the complexity of regulatory landscapes [23]. In this work, we apply our GP-GCN framework to the regulatory item identification task.

To collect the sequences from enhancers and promoters, we download the human reference genome hg38 and identify the locations of the regulatory elements from Ensembl (`https://ftp.ensembl.org`). We randomly extract 10,000 sequences from enhancer, promoter, and non-regulatory regions, respectively.

The GP-GCN framework obtains an accuracy of 0.839 and a macro F1-score of 0.840 for identifying enhancers and promoters from the genome. With accuracy increased by 1.5-45.2% and macro F1-score increased by 2.5-57.2% compared with alternative models, the results further highlight the superiority of our GP-GCN framework (Supplementary Table S18).

S2.4.3 lncRNA localization prediction

Long non-coding RNAs (lncRNAs) are a class of RNA molecules that play diverse roles in cellular processes, including gene expression regulation, chromatin remodeling, and epigenetic modification [24]. The subcellular localization of lncRNAs, denoting their precise localization within cellular compartments or organelles, presents a spectrum of possibilities, encompassing the nucleus, cytoplasm, ribosomes, exosomes, and other cellular locales [25]. Subcellular localization of lncRNAs is crucial for comprehending their function in cellular processes and biological pathways.

For the lncRNA localization prediction task, we applied the 769 lncRNA sequences from four subcellular localizations, namely cytoplasm, exosome, nucleus, and ribosome, which are collected from RNALocate database [26, 25].

Our proposed GP-GCN framework outperformed GraphLncLoc [25] and other models by achieving an accuracy of 0.664 and a macro F1-score of 0.566, showing a remarkable performance increase, with 5.4-22.6% in accuracy and 6.4-40.1% in macro F1-score, for predicting the subcellular localization of lncRNAs (Supplementary Table S19).

S2.5 Performance of GP-GCN framework on sequences with various lengths

Among the seven downstream tasks encompassed in this work, the phage integration site prediction task operates on sequences with a fixed length (600*bp*), whereas the input sequence lengths vary considerably for the other six tasks, phage and ICE discrimination, phage lifestyle prediction, phage host prediction, species classification with MHC, regulatory item identification, and lncRNA localization prediction (Supplementary Figures S5 and S16). To further validate the performance of the GP-GCN framework across sequences with diverse lengths, we partition the test sequences into distinct length intervals for each of these six tasks. Subsequently, we separately evaluated the performance of the trained models on sequences corresponding to each length interval. The results demonstrate that our model exhibits consistent and reliable performance across sequences with various lengths for these six tasks (Supplementary Figure S17).

S2.6 Mining gapped patterns of more significant influence in prediction tasks

From the models of Graphage, we calculate the contribution scores for the patterns and pattern groups (see *Contribution score of gapped patterns and motifs* subsection) to mine informative patterns for the phage-related tasks.

For phage lifestyle prediction, we present the contribution score distribution for the gapped patterns in Supplementary Figure S19 A. The bimodal distribution shows that some patterns have relatively high contribution scores (0.15-0.4). In Supplementary Figure S19 B, we give the occurrence frequencies for the five gapped patterns with the highest contribution scores (left) and the frequencies for the five with the lowest contribution scores (right). We note that the gapped patterns with the highest contribution scores demonstrate significantly different frequencies between temperate and virulent phages, suggesting their significance in the classification task. We also analyze the contribution scores of the pattern groups (Supplementary Figure S19 C). The results also indicate that patterns with high contribution scores are more likely to occur in temperate phages.

For the phage and ICE discrimination task and integration site prediction task, the contribution score distribution is unimodal (Supplementary Figure S20 left), with the peak <0.05, indicating that the significance of a single gapped pattern is unremarkable. However, the analyses for the pattern groups (Supplementary Figure S20 middle and right) show that for phage and ICE discrimination, the patterns with high contribution scores are more likely to occur in ICE sequences; for integration site prediction, the patterns with high contribution scores are more likely to occur in the integration sites.

S2.7 Transcription factor associations at phage and bacterial integration sites

The identified transcription factors (TFs) were reported to be associated with viral activities. Specifically, for the phage integration site, the motif "GATTTTTGCT" matches with RpoN. RpoN was reported to participate in signal-response mechanisms for activities such as motility and virulence ([27]). Also, the motif "ATATCACA" can be aligned to CRP, which is involved in phage replication ([28]). For the bacterial integration site, the motif "ATGGAAGGCGCKATC" is consistent with CcpA, which is down-regulated after viral infection ([29]). The motif "AMAAMWCA" matches ToxT, which is found in the region containing a putative integrase ([30]).

S3 Supplementary figures

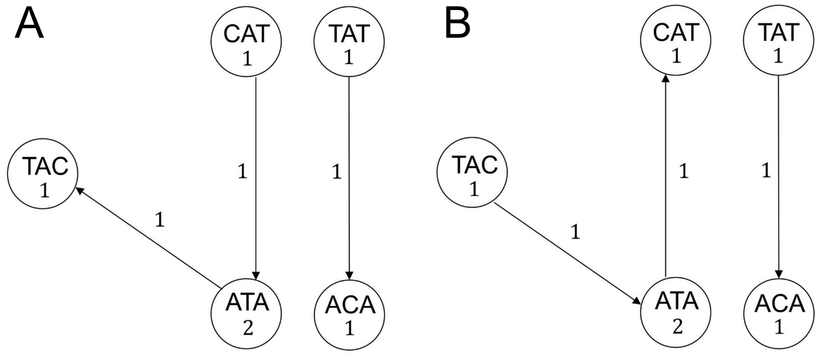

Figure S1: Examples of pattern graph construction.
A. $G(\text{CATATACA}, 3)$, the 3-mer graph for the string CATATACA. B. $G(\text{TATACATA}, 3)$, the 3-mer graph for the string TATACATA. The two strings result in the same 3-mer distribution but different 3-mer graphs. The vertices and edges with a feature value of zero are not displayed in the examples, despite their presence within the constructed graph.

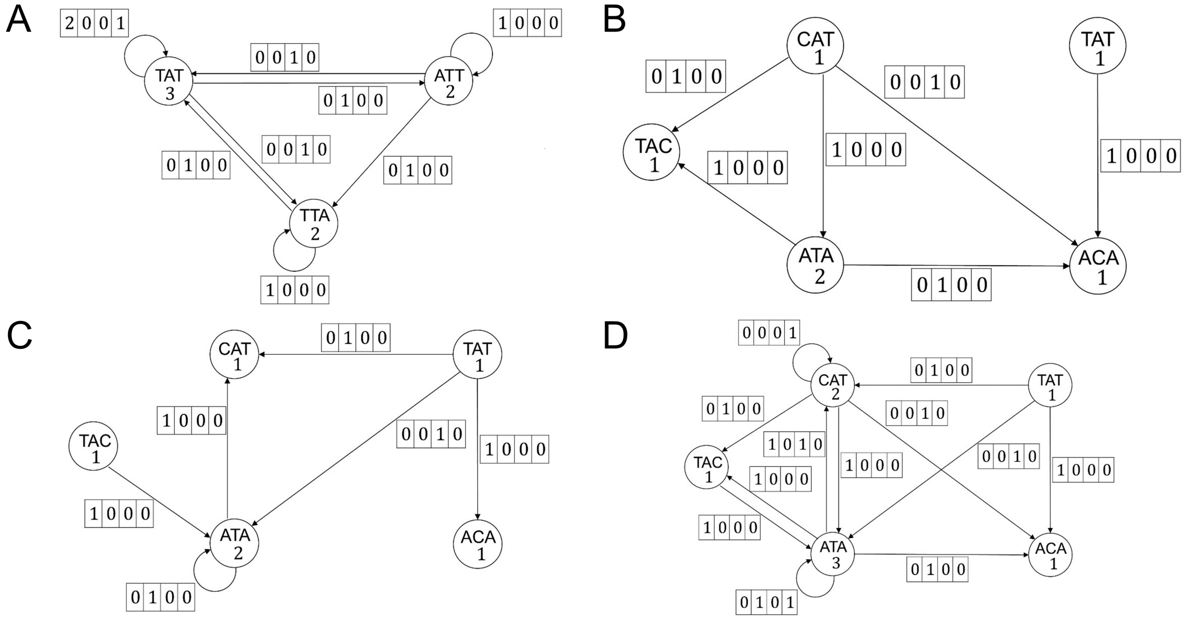

Figure S2: Examples of gapped pattern graph construction.
A. G(TATTATTAT,3,3), the gapped 3-mer graph for the string TATTATTAT which allows up to a gap of length 3. B. G(CATATACA, 3, 3), the gapped 3-mer graph for the string CATATACA which allows up to a gap of length 3. C. G(TATACATA, 3, 3), the gapped 3-mer graph for the string TATACATA which allows up to a gap of length 3. D. G(CATATACATA, 3, 3), the gapped 3-mer graph for the string CATATACATA which allows up to a gap of length 3. The vertices with a feature value of 0 and edges with a feature vector of {0,0,0,0} are not displayed in the examples, despite their presence within the constructed graph.

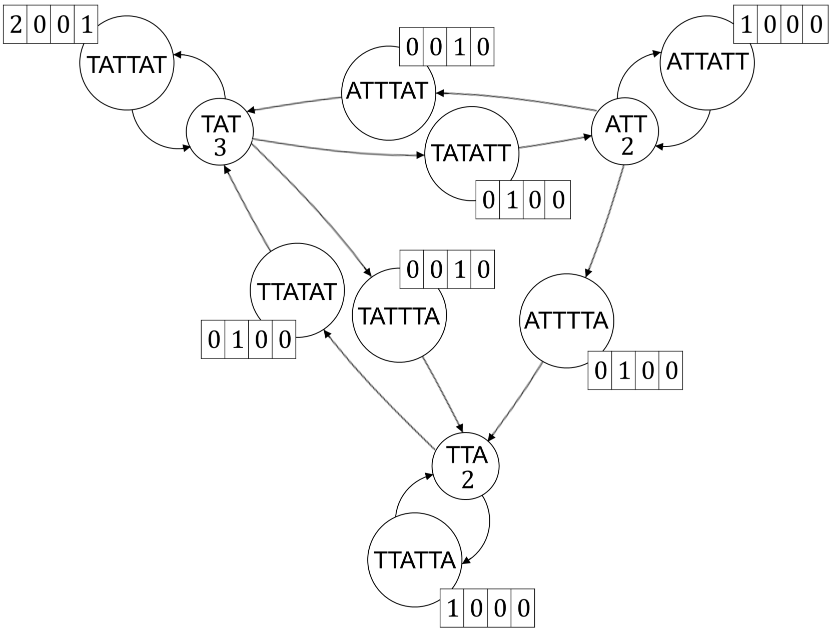

Figure S3: An example of bipartite pattern graph construction.
S(TATTATTAT,3,3), the gapped 3-mer graph for the string TATTATTAT which allows up to a gap of length 3, after conversion of edges into vertices. Each vertex contains a (featureless) self-loop for GCN computation, which is not shown in the figure. The vertices with a feature of 0 or {0,0,0,0} are not displayed in the examples, despite their presence within the constructed graph.

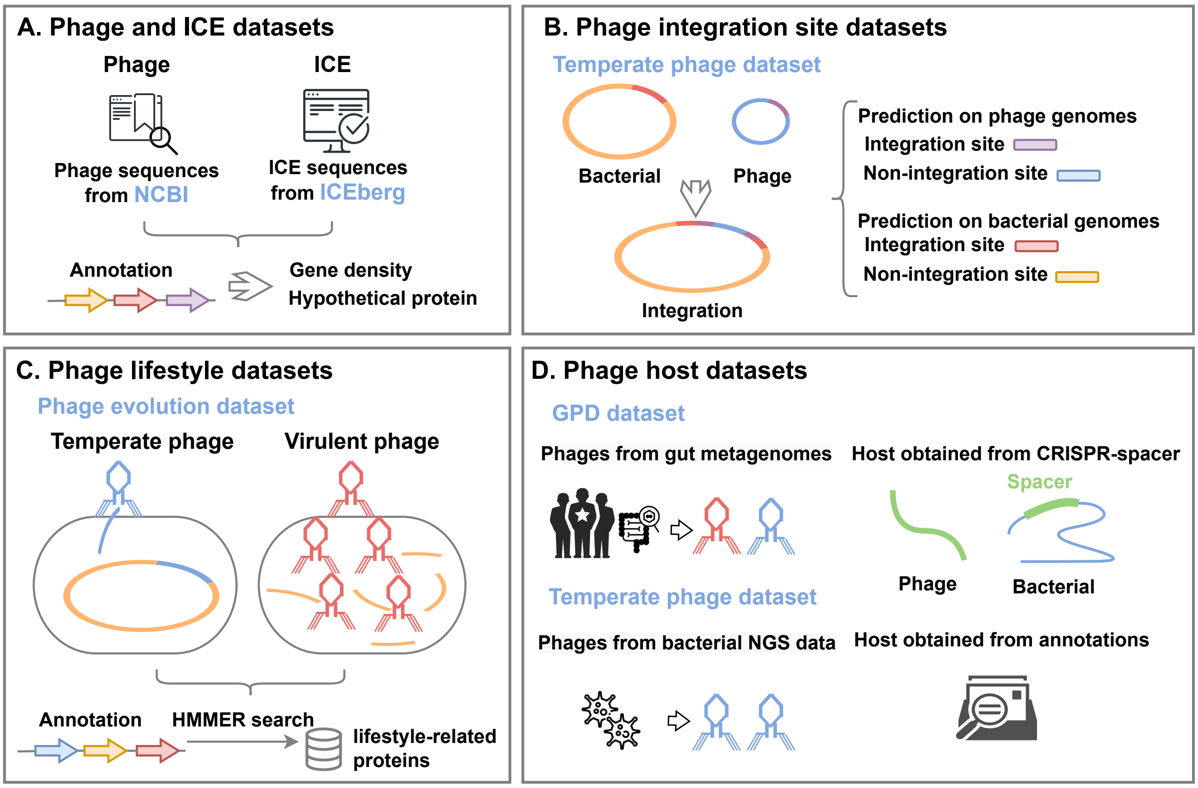

Figure S4: Four benchmark datasets are prepared, each for a phage-related task. A. Phage and ICE discrimination. B. Phage integration site prediction (on phage and bacterial genomes, respectively). C. Phage lifestyle prediction. D. Phage host prediction.

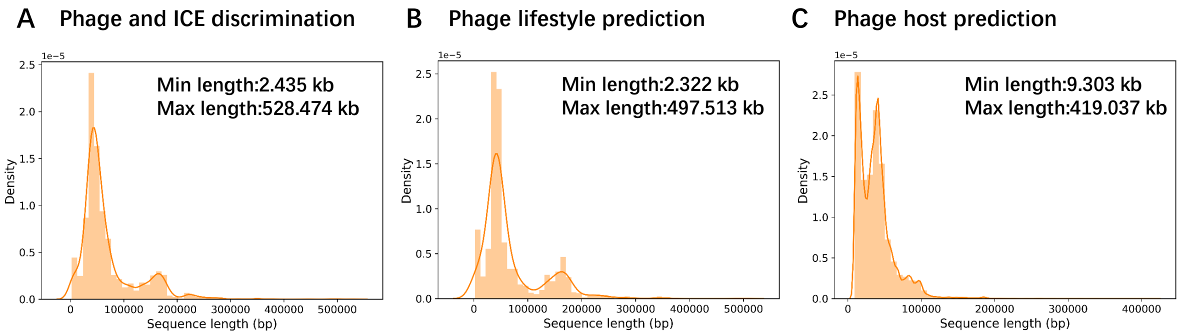

Figure S5: The length distributions of the input sequences for the phage-related tasks. The input sequence length distributions of A. phage and ICE discrimination, B. phage lifestyle prediction, and C. phage host prediction. The sequences in phage integration site datasets have a consistent length of 600 *bp*.

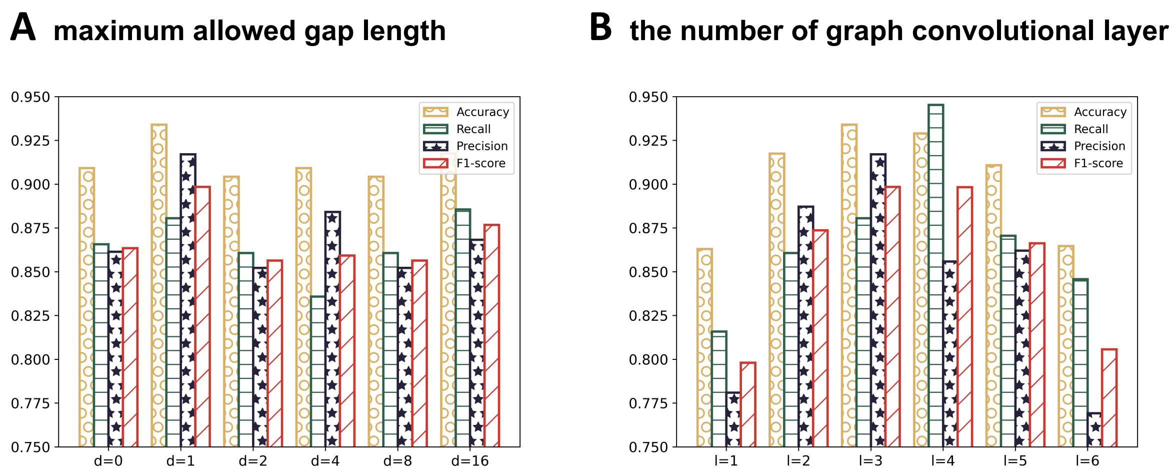

Figure S6: The effect of hyperparameters on the performance of phage and ICE discrimination. The effect of (A) maximum allowed gap length (d), and (B) the number of graph convolutional layers (l) on the performance of phage and ICE discrimination. (Ranges of gap lengths were used for d larger than 2.)

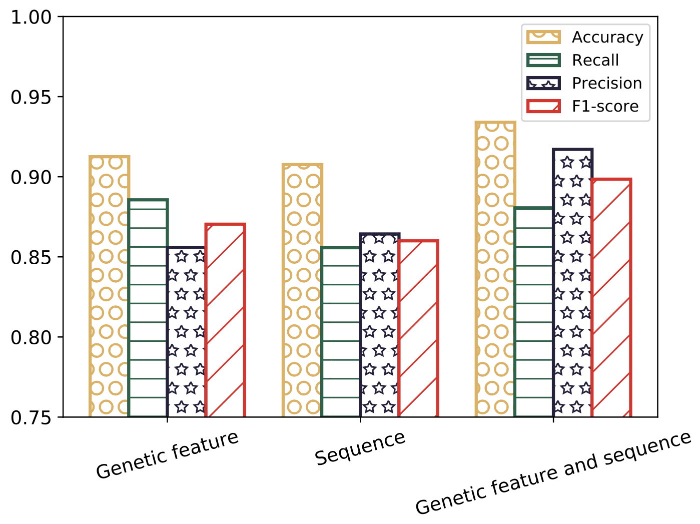

Figure S7: The effect of input features on the performance of phage and ICE discrimination. The phage and ICE discrimination performance of models with only sequences, only genetic features, and both sequences and genetic features as inputs.

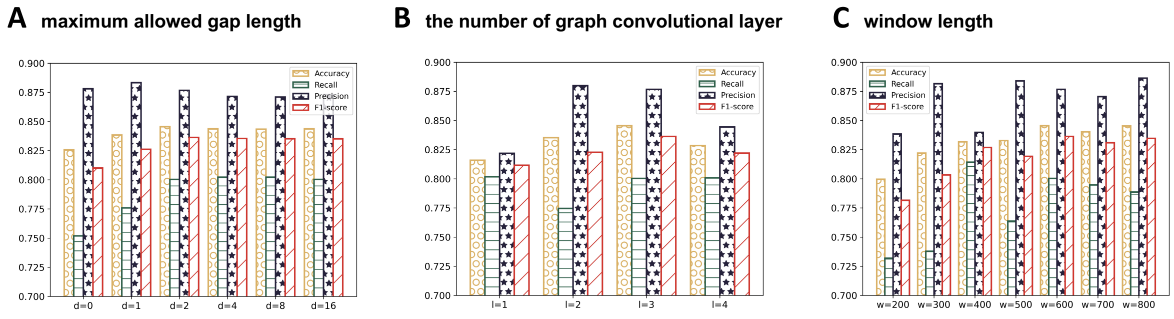

Figure S8: The effect of hyperparameters on the performance of integration site prediction on phage genomes.

The effect of (A) maximum allowed gap length (d), (B) the number of graph convolutional layers (l), and (C) window length (w) on the performance of integration site prediction on phage genomes. (Ranges of gap lengths were used for d larger than 2.)

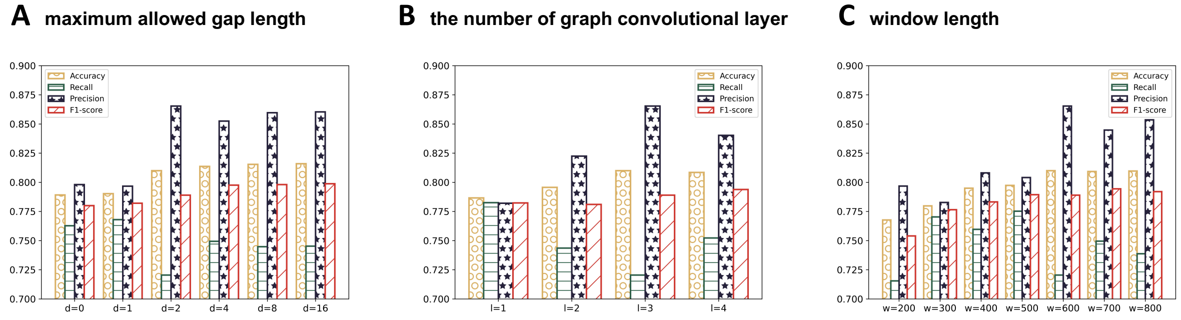

Figure S9: The effect of hyperparameters on the performance of integration site prediction on bacteria genomes.

The effect of (A) maximum allowed gap length (d), (B) the number of graph convolutional layers (l), and (C) window length (w) on the performance of integration site prediction on bacteria genomes. (Ranges of gap lengths were used for d larger than 2.)

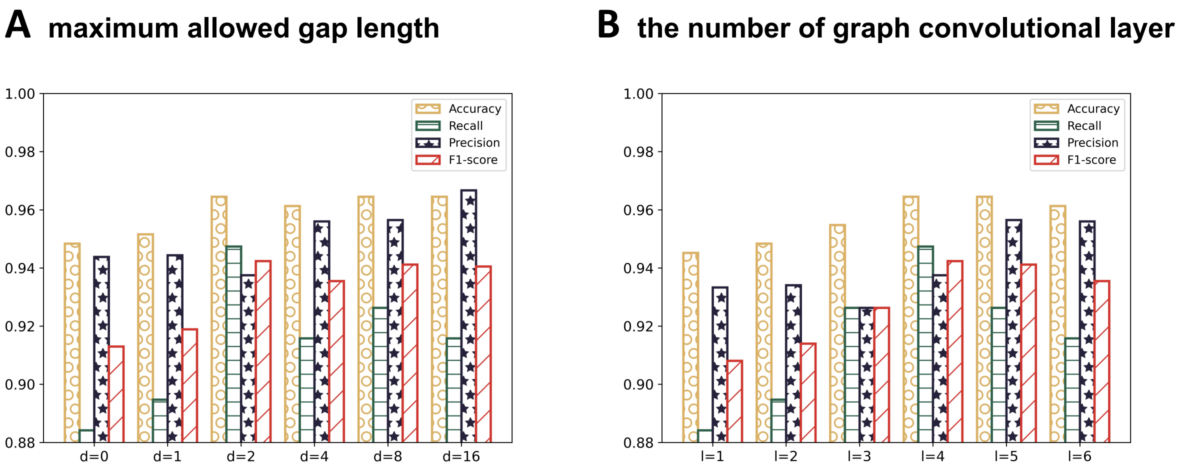

Figure S10: The effect of hyperparameters on the performance of phage lifestyle prediction. The effect of (A) maximum allowed gap length (d) and (B) the number of graph convolutional layers (l) on the performance of phage lifestyle prediction. (Ranges of gap lengths were used for d larger than 2.)

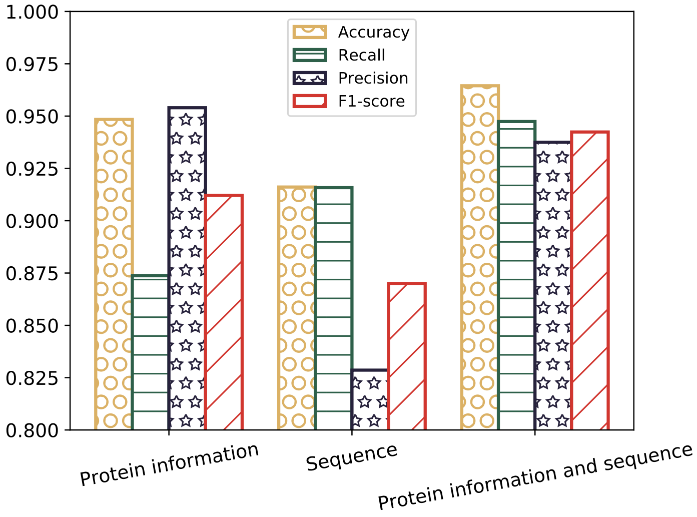

Figure S11: The effect of input features on the performance of lifestyle prediction. The lifestyle prediction performance of models with only sequences, only genetic features, and both sequences and genetic features as inputs.

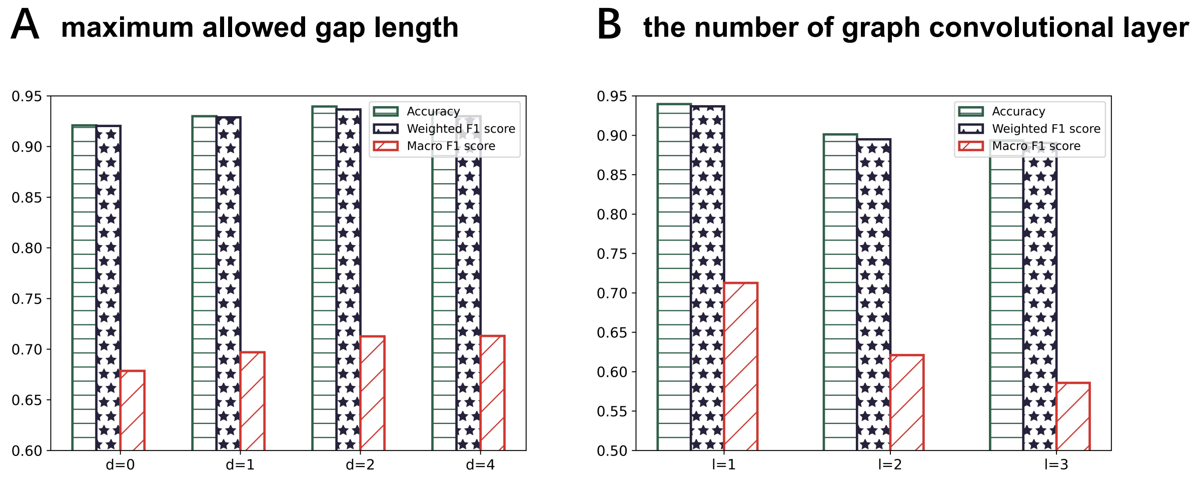

Figure S12: The effect of hyperparameters on the performance of phage host prediction. The effect of (A) maximum allowed gap length (d) and (B) the number of graph convolutional layers (l) on the performance of phage host prediction. (Ranges of gap lengths were used for d larger than 2.)

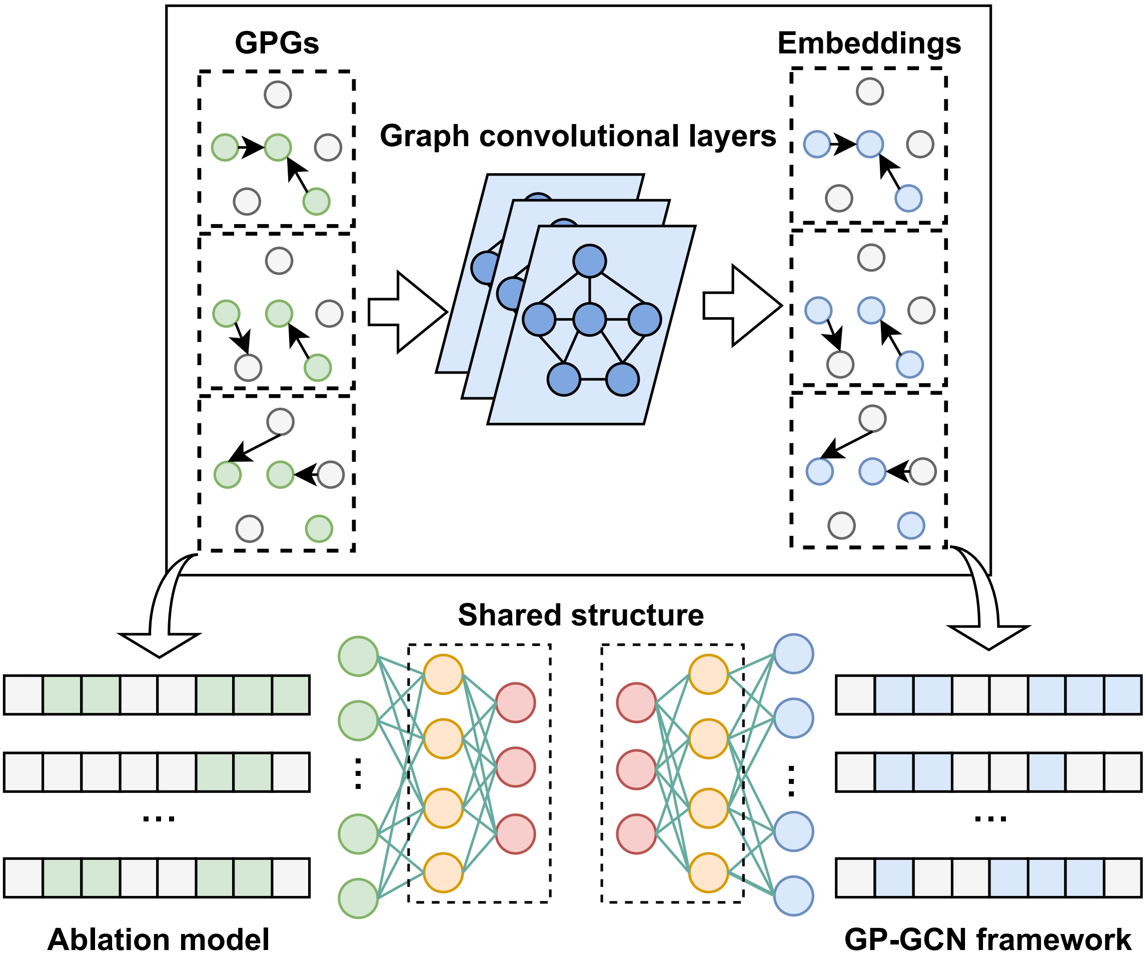

Figure S13: Illustration of the ablation model.
In the ablation model, the gapped pattern frequencies from the GPGs are directly concatenated and fed into a fully-connected neural network; In our GP-GCN framework, these gapped pattern frequencies are passed through the graph convolutional layers to generate embeddings before being fed into a fully-connected neural network sharing the same structure.

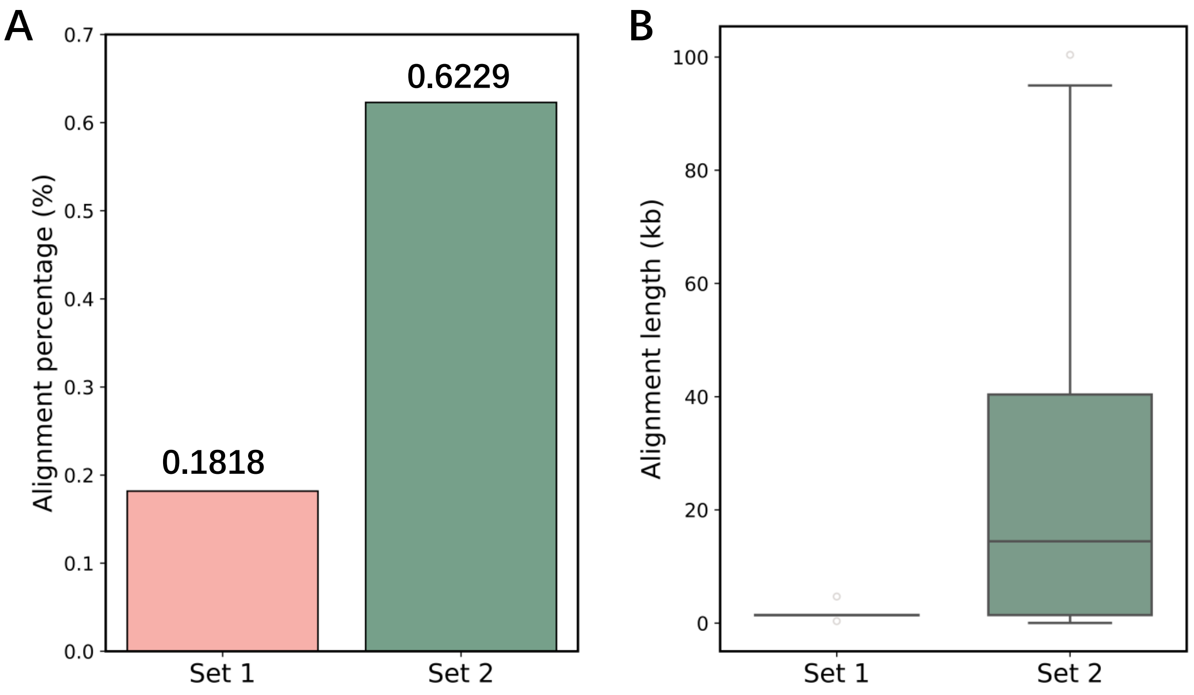

Figure S14: Analysis of the sequences that could be correctly classified by Graphage but not by the MLP model.

For the phage and ICE discrimination task, we gathered two sets of sequences. Set 1 was comprised of sequences that could be correctly classified by Graphage but not by the MLP model. Set 2 was composed of sequences that could be correctly classified by both Graphage and MLP. Aligning the two sets of sequences to the training set, we show the alignment percentage (A) and alignment length (B).

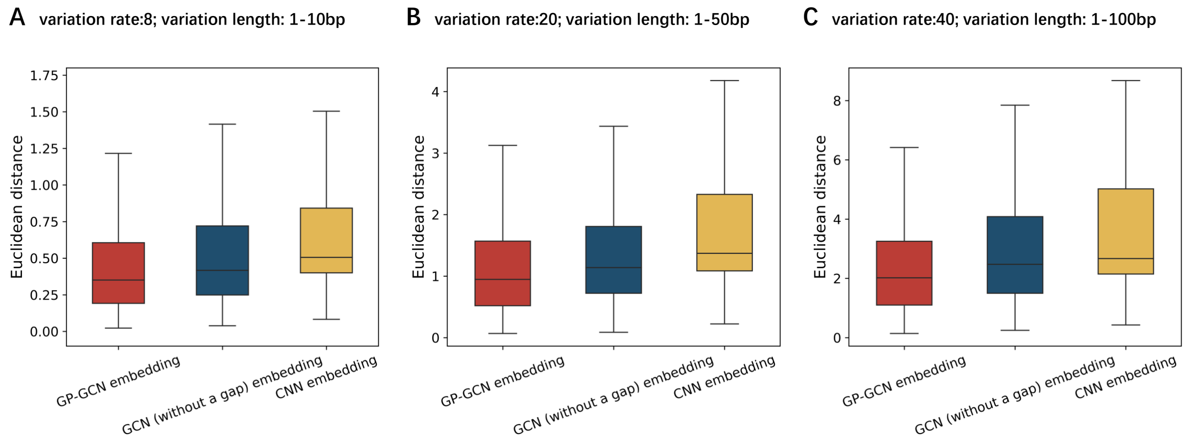

Figure S15: The distance between the vectors of the original sequences and mutated sequences from different embedding.

For the three simulated datasets, we calculated the Euclidean distance between the vectors of the original sequences and mutated sequences, from the GCN-produced embeddings, GCN (without a gap)-produced embeddings and CNN-produced embeddings.

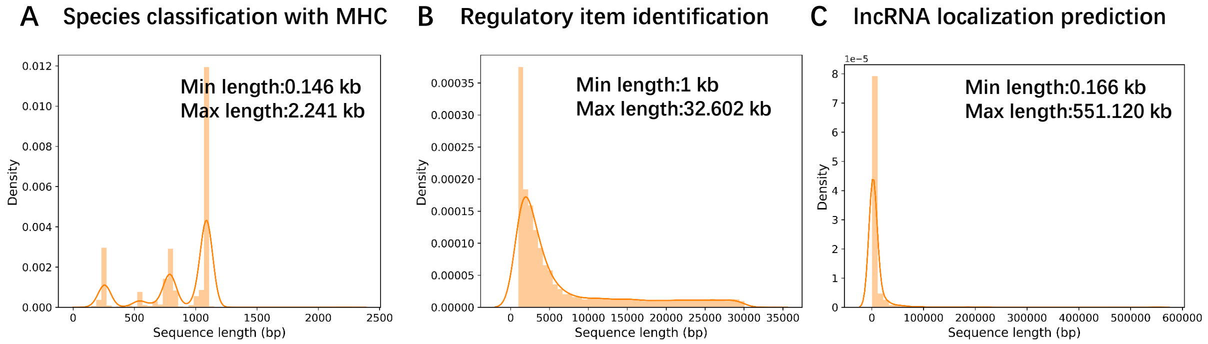

Figure S16: The length distributions of the input sequences for the three extended applications. The input sequence length distributions of A. species classification with MHC, B. regulatory item identification, and C. lncRNAs localization prediction.

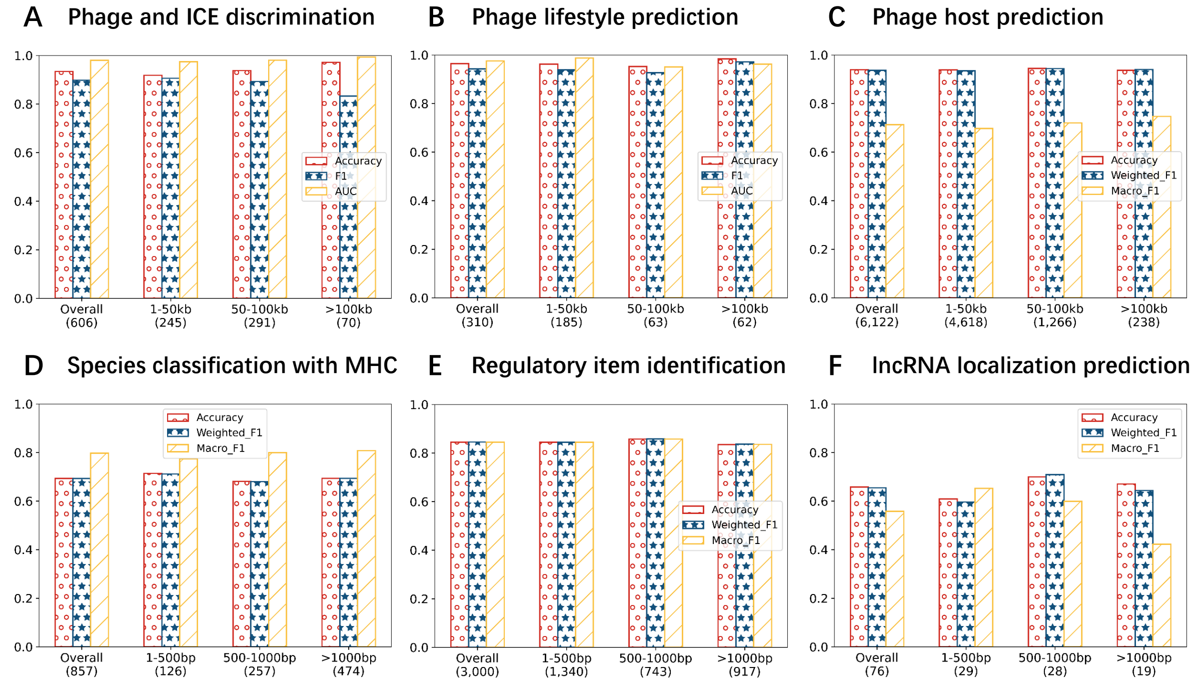

Figure S17: Model performance on sequences with variable lengths.
The performance of the GP-GCN framework on A. phage and ICE discrimination, B. phage lifestyle prediction, C. phage host prediction, D. species classification with MHC, E. regulatory item identification, and F. lncRNAs localization prediction, evaluated on the test sequences within different length intervals. The numbers in parentheses represent the number of sequences within the interval range.

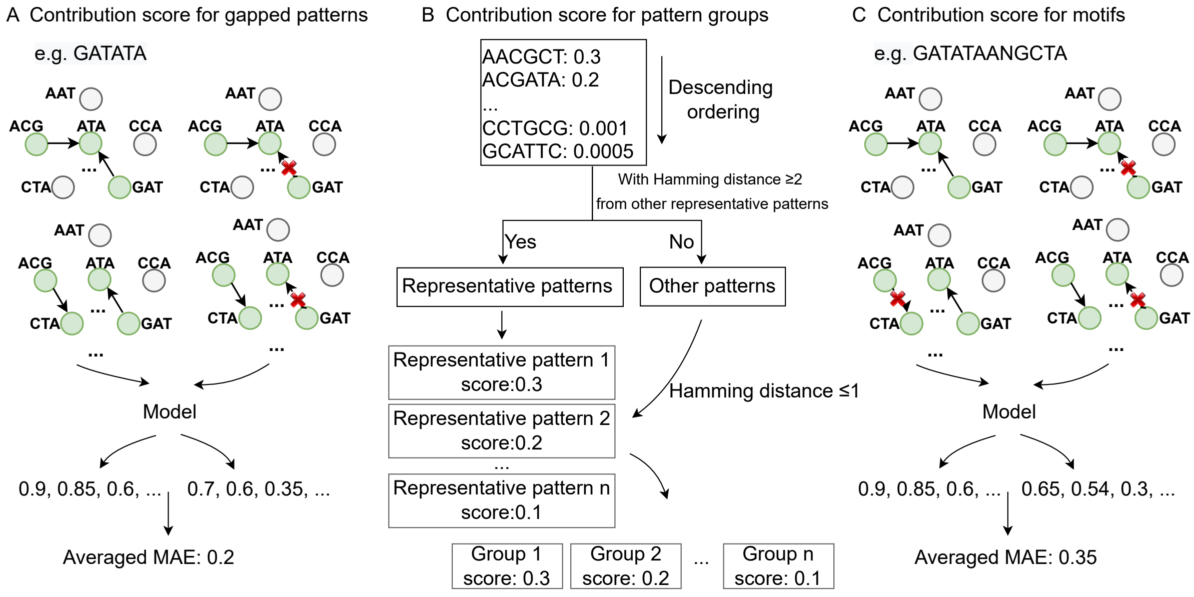

Figure S18: Contribution score calculation.
The calculation of contribution score for gapped patterns (A), pattern groups (B), and motifs (C).

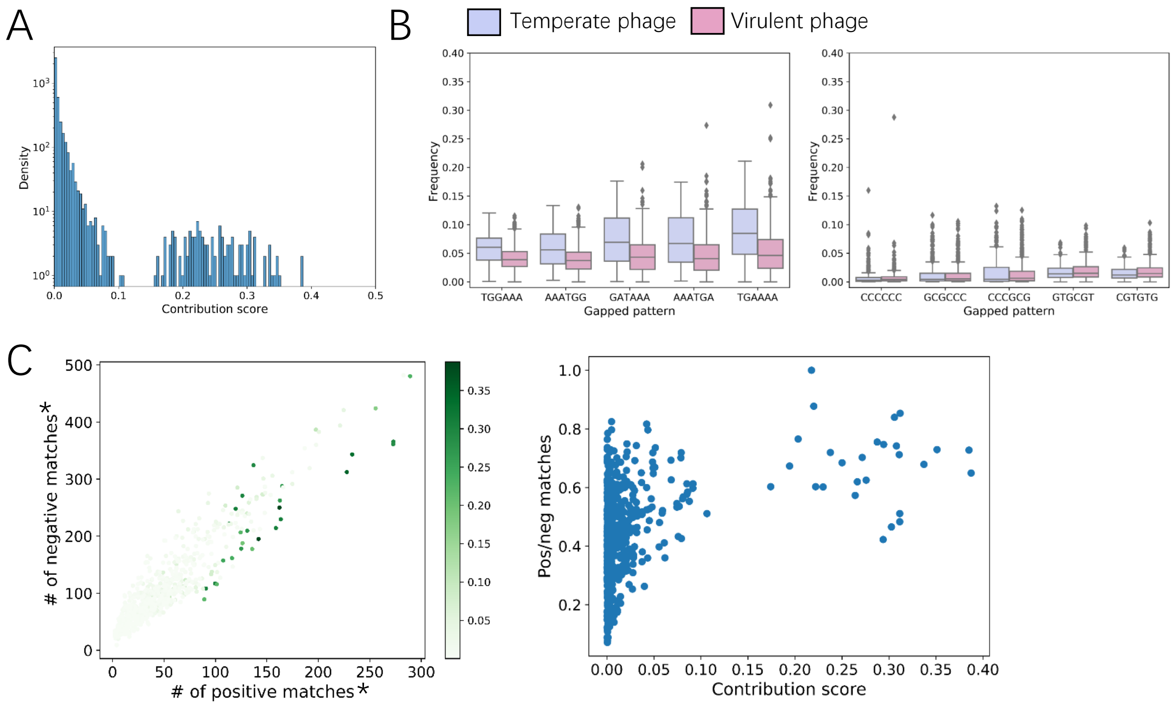

Figure S19: The contribution score analysis of the gapped patterns for phage lifestyle prediction. A. The contribution score distribution for the 4,096 gapped patterns to the phage lifestyle prediction. B. The occurrence frequencies for the five gapped patterns with the highest (left) and lowest (right) contribution scores in temperate phages and virulent phages. C. The number of positive matches and negative matches for each gapped pattern group, with the color indicating the contribution score (left). The ratio of positive matches to negative matches and contribution score for each gapped pattern group (right). *The number of positive (negative) matches is the number of positive (negative) sequences that contain the pattern. For each sequence, we only count the 10% highest matching patterns. For phage lifestyle prediction, positive sequences are temperate phages, while negative sequences are virulent phages.

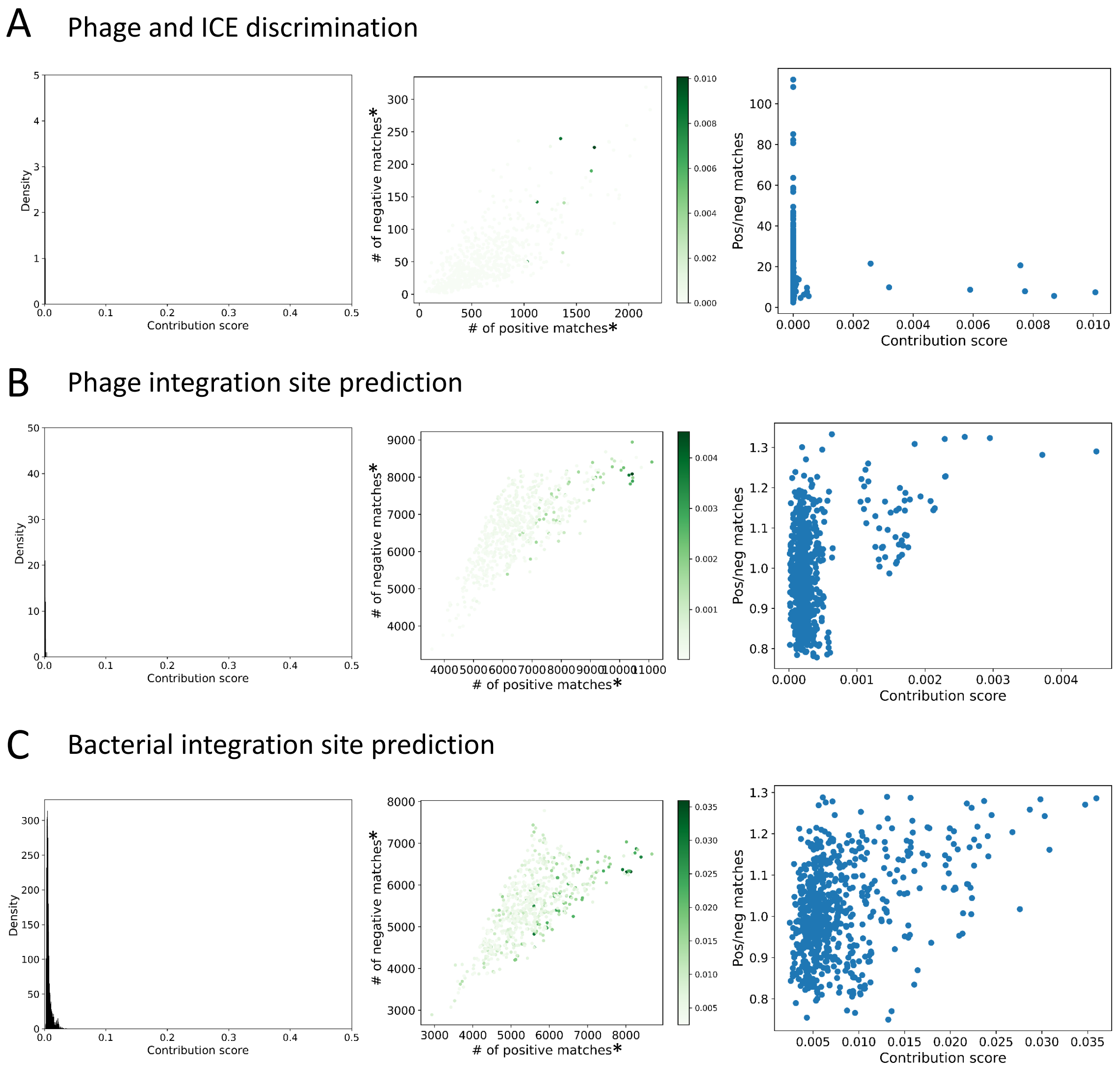

Figure S20: The contribution score analysis of the gapped patterns for phage and ICE discrimination, phage integration site prediction, and bacterial integration site prediction.

For A. phage and ICE discrimination, B. phage integration site prediction, and C. bacterial integration site prediction, we show the contribution score distribution for the 4,096 gapped patterns (left); For each gapped pattern group, we show the number of positive matches, the number of negative matches, and the contribution score (middle and right). *For phage and ICE discrimination, positive sequences are phages, while negative sequences are ICEs. For phage and bacterial integration site prediction, positive sequences are integration sites, while negative sequences are non-integration sites.

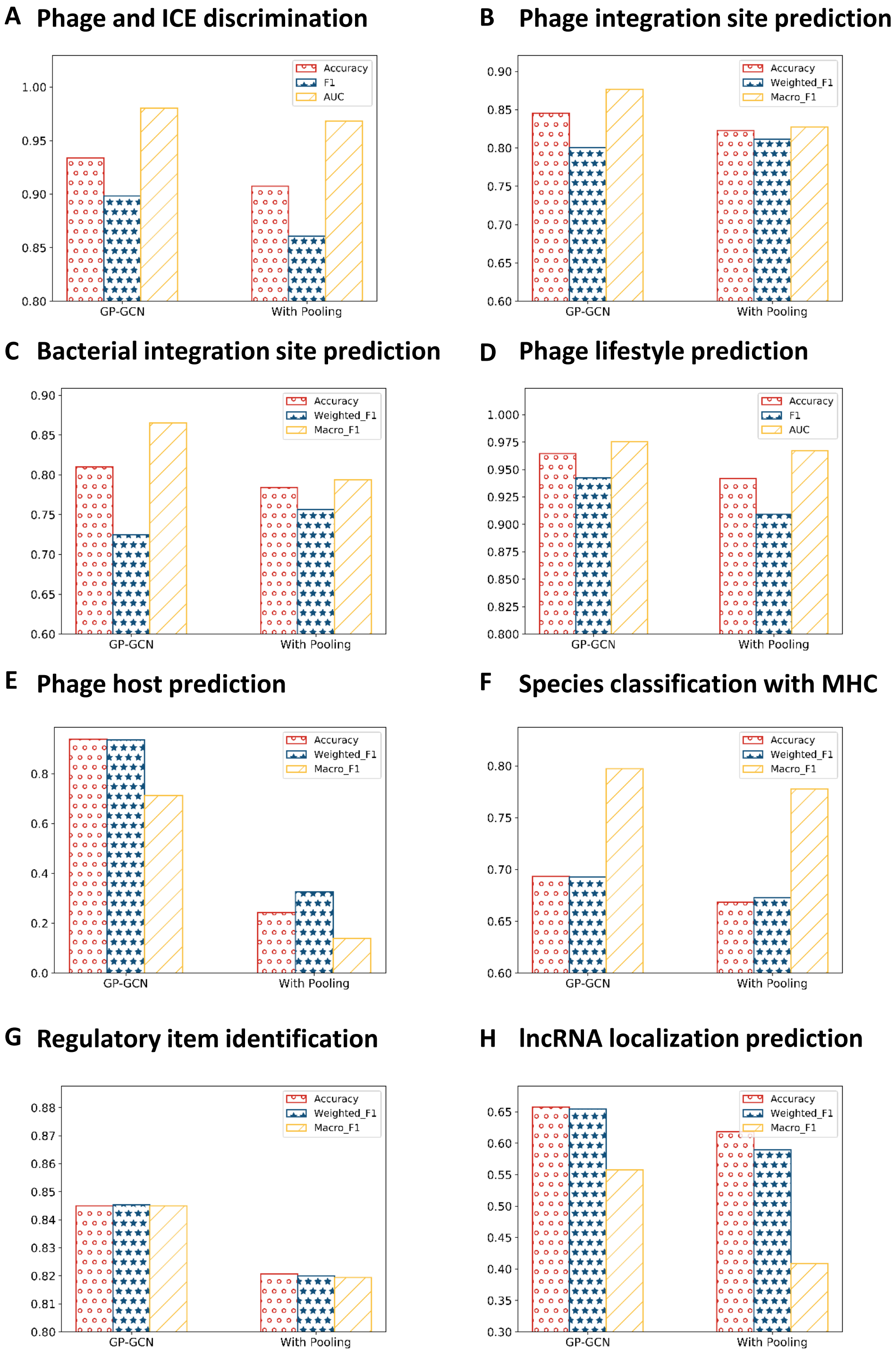

Figure S21: Performance comparison of the models from the original GP-GCN framework and the models when edge embeddings from the final graph convolutional layer are average pooled.
For the eight prediction tasks studied in this work, we compare the performance of the models from the original GP-GCN framework and the models when edge embeddings are average pooled with global mean pool function in the torch-geometric package.

S4 Supplementary tables

Parameter type	Parameter	Parameter setting	Description
Model parameters	label_num	2	The number of labels.
	other_feature_dim	2	The dimension for other features.
	K	3	The length of k-mer.
	d_n	{0,1}	The gap lengths allowed in gapped patterns.
	node_hidden_dim	3	$\|h_v^{l+1}\|$.
	gcn_dim	128	$\|h_u^{l+1}\|$; The dimension of GCN embeddings.
	gcn_layer_num	3	The number of GCN layers.
	cnn_dim	64	The dimension of convolutional layer embeddings.
	cnn_layer_num	3	The number of convolutional layers.
	cnn_kernel_size	8	The kernel size of convolutional layers.
	fc_dim	100	The dimension of fully connected layer embeddings.
	dropout_rate	0.2	The dropout rate.
	pnode_nn	Yes	Whether to embed primary features into latent space.
	fnode_nn	Yes	Whether to embed target features into latent space.
Training parameters	learning_rate	1e-4	The learning rate for training.
	batch_size	64	The batch size for training.
	epoch_n	100	The number of training epochs.
	val_split	0.1	The validation set size.

Table S1: The hyperparameters used for phage and ICE discrimination in Graphage.

Parameter type	Parameter	Parameter setting	Description
Model parameters	label_num	2	The number of labels.
	other_feature_dim	0	The dimension for other features.
	K	3	The length of k-mer.
	d_n	{0,1,2}	The gap lengths allowed in gapped patterns.
	node_hidden_dim	3	$\|h_v^{l+1}\|$.
	gcn_dim	128	$\|h_u^{l+1}\|$; The dimension of GCN embeddings.
	gcn_layer_num	3	The number of GCN layers.
	cnn_dim	64	The dimension of convolutional layer embeddings.
	cnn_layer_num	3	The number of convolutional layers.
	cnn_kernel_size	8	The kernel size of convolutional layers.
	fc_dim	100	The dimension of fully connected layer embeddings.
	dropout_rate	0.2	The dropout rate.
	pnode_nn	Yes	Whether to embed primary features into latent space.
	fnode_nn	Yes	Whether to embed target features into latent space.
Training parameters	learning_rate	1e-4	The learning rate for training.
	batch_size	32	The batch size for training.
	epoch_n	50	The number of training epochs.
	val_split	0.1	The validation set size.

Table S2: The hyperparameters used for integration site prediction in Graphage.

Parameter type	Parameter	Parameter setting	Description
Model parameters	label_num	2	The number of labels.
	other_feature_dim	206	The dimension for other features.
	K	3	The length of k-mer.
	d_n	{0,1,2}	The gap lengths allowed in gapped patterns.
	node_hidden_dim	3	$\|h_v^{l+1}\|$.
	gcn_dim	128	$\|h_u^{l+1}\|$; The dimension of GCN embeddings.
	gcn_layer_num	4	The number of GCN layers.
	cnn_dim	64	The dimension of convolutional layer embeddings.
	cnn_layer_num	3	The number of convolutional layers.
	cnn_kernel_size	8	The kernel size of convolutional layers.
	fc_dim	100	The dimension of fully connected layer embeddings.
	dropout_rate	0.2	The dropout rate.
	pnode_nn	Yes	Whether to embed primary features into latent space.
	fnode_nn	Yes	Whether to embed target features into latent space.
Training parameters	learning_rate	1e-4	The learning rate for training.
	batch_size	64	The batch size for training.
	epoch_n	200	The number of training epochs.
	val_split	0.1	The validation set size.

Table S3: The hyperparameters used for phage lifestyle prediction in Graphage.

Parameter type	Parameter	Parameter setting	Description
Model parameters	label_num	107	The number of labels.
	other_feature_dim	0	The dimension for other features.
	K	3	The length of k-mer.
	d_n	{0,1,2}	The gap lengths allowed in gapped patterns..
	node_hidden_dim	3	$\|h_v^{l+1}\|$.
	gcn_dim	100	$\|h_u^{l+1}\|$; The dimension of GCN embeddings.
	gcn_layer_num	1	The number of GCN layers.
	cnn_dim	100	The dimension of convolutional layer embeddings.
	cnn_layer_num	2	The number of convolutional layers.
	cnn_kernel_size	2	The kernel size of convolutional layers.
	fc_dim	500	The dimension of fully connected layer embeddings.
	dropout_rate	0	The dropout rate.
	pnode_nn	No	Whether to embed primary features into latent space.
	fnode_nn	Yes	Whether to embed target features into latent space.
Training parameters	learning_rate	1e-4	The learning rate for training.
	batch_size	128	The batch size for training.
	epoch_n	2000	The number of training epochs.
	val_split	0.1	The validation set size.

Table S4: The hyperparameters used for phage host prediction in Graphage.

**Task**	# of training samples	# of validation samples	# of test samples	# of total samples
Phage and ICE discrimination	5,418	602	606	6,626
Phage integration site prediction	33,788	4,223	4,223	42,234
Bacterial integration site prediction	28,080	3,510	3,510	35,100
Phage lifestyle prediction	1,241	155	155	1,551
Phage host prediction	4,8973	6,122	6,122	61,217
Species classification with MHC	6,860	858	858	8,576
Regulatory item identification	24,000	3,000	3,000	30,000
lncRNA localization prediction	615	77	77	769

Table S5: The number of samples in training, validation, and test sets for each task.

	Model	Accept variable length inputs	Model k-mer interactions	Tolerate genomic variations	Applied sequences
GP-GCN (Ours)	GCN	✓	✓	✓	Microbe genomes<br>MHC genes<br>Regulatory elements<br>lncRNAs
AF-based	Vector distance	✓	×	×	-
word2vec-based	CBOW	✓	×	×	-
Sequence-based	CNN	×	✓	×	-
Nucleotide Transformer	Transformer	✓*	✓	×	Human genomes<br>Multi-species genomes
DNABERT	Transformer	✓*	✓	×	Human genomes
DNA-GCN	GCN	✓	✓	×	TF-binding sites
GraphLncLoc	GCN	✓	✓	×	lncRNAs

Table S6: Feature and application comparisons for the genome encoding models.*Nucleotide Transformer and DNABERT accept DNA sequences with maximum lengths of 512 *bp* and 6 *kb*, respectively.

Task	Dataset	# of sequences	# of classification
Phage and ICE discrimination	NCBI [1] and ICEBerg [2]	6,020	2
	ImmeDB [4]	606	2
	Total	6,626	2
Phage integration site prediction	integration sites on phages [6]	42,234	2
	integration sites on bacteria [6]	35,100	2
Phage lifestyle prediction	Phage evolution dataset [7]	1,551	2
Phage host prediction	GPD [3]	1,071	37
	Temperate phage dataset [6]	60,146	104
	Total	61,217	107

Table S7: Summary of benchmark datasets in this study.

Method*	Accuracy	F1 score	AUC
Graphage (Ours)	**0.9340**	**0.8985**	**0.9805**
AF-based	0.8564	0.7629	-
word2vec-based	0.8614	0.7868	0.9105
Sequence-based	0.3317	0.4981	0.5487
Gapped pattern frequency-based using			
k-Nearest Neighbors	0.6683	0.6516	0.8977
Random Forest	0.3333	0.4987	0.6873
MLP	0.9109	0.8492	0.9773
GPD [3]	0.5924	0.6159	0.9624

Table S8: Comparing Graphage with other models/tools on phage and ICE discrimination tasks. The best-performing model is in bold font, while the runner-up is underlined. *AUC is inapplicable for the AF-based model since the model does not give probabilities.

Method	Accuracy	F1 score	AUC
Graphage (Ours)	**0.849±0.005**	**0.839±0.006**	**0.882±0.004**
AF-based	0.810±0.004	0.812±0.004	-
word2vec	0.791±0.007	0.779±0.010	0.844±0.008
Sequence-based	0.736±0.013	0.734±0.007	0.809±0.007
Nucleotide Transformer	0.806±0.007	0.795±0.008	0.807±0.007
DNABERT	0.822±0.005	0.819±0.004	0.866±0.005
Gapped pattern frequency-based using			
k-Nearest Neighbors	0.690±0.006	0.741±0.006	0.801±0.006
Random Forest	0.802±0.008	0.786±0.006	0.874±0.002
MLP	0.805±0.006	0.797±0.006	0.854±0.004

Table S9: Comparing our graph model with other models/tools on phage integration site prediction tasks.
The best performing model is in bold font, while the runner-up is underlined. Results are averaged over ten random train-test splits.

Method	Accuracy	F1 score	AUC
Graphage (Ours)	**0.813±0.005**	**0.801±0.007**	**0.860±0.006**
AF-based	0.772±0.007	0.782±0.008	-
word2vec	0.769±0.006	0.763±0.006	0.836±0.006
Sequence-based	0.735±0.010	0.732±0.012	0.820±0.012
Nucleotide Transformer	0.781±0.009	0.780±0.015	0.783±0.011
DNABERT	0.788±0.006	0.787±0.005	0.852±0.004
Gapped pattern frequency-based using			
k-Nearest Neighbors	0.588±0.031	0.690±0.006	0.681±0.008
Random Forest	0.785±0.021	0.767±0.031	0.855±0.007
MLP	0.774±0.006	0.769±0.007	0.833±0.007

Table S10: Comparing our graph model with other models and tools on bacterial integration site prediction tasks.
The best performing model is in bold font, while the runner-up is underlined. Results are averaged over ten random train-test splits.

Method	Accuracy	F1 score	AUC
Graphage (Ours)	**0.957±0.011**	**0.928±0.022**	**0.975±0.010**
AF-based	0.894±0.014	0.836±0.310	-
word2vec	0.939±0.011	0.898±0.021	0.959±0.009
Sequence-based	0.810±0.047	0.657±0.064	0.858±0.023
Gapped pattern frequency-based using			
k-Nearest Neighbors	0.875±0.023	0.803±0.044	0.926±0.016
Random Forest	0.890±0.018	0.805±0.040	0.948±0.019
MLP	0.938±0.017	0.895±0.035	0.967±0.011
BACPHLIP [8]	0.932±0.007	0.888±0.016	0.956±0.009
DeePhage [15]	0.845±0.030	0.875±0.024	0.969±0.010

Table S11: Comparing our graph model with other models and tools on phage lifestyle prediction tasks. The best performing model is in bold font, while the runner-up is underlined. Results are averaged over ten random train-test splits.

Method	Accuracy	Weighted F1 score	Macro F1 score
Graphage (Ours)	**0.940±0.001**	**0.937±0.001**	**0.730±0.024**
AF-based	0.931±0.001	0.931±0.001	0.715±0.001
word2vec	0.746±0.032	0.725±0.033	0.358±0.014
Sequence-based	0.644±0.035	0.610±0.051	0.210±0.033
Gapped pattern frequency-based using			
k-Nearest Neighbors	0.935±0.003	0.932±0.002	0.715±0.004
Random Forest	0.923±0.003	0.913±0.003	0.679±0.019
MLP	0.798±0.056	0.430±0.048	0.682±0.074
HostPhinder [16]	0.320±0.010	0.416±0.017	0.136±0.028
VirHostMatcher [17]	0.380±0.005	0.372±0.005	0.461±0.011
WIsH [17]	0.377±0.007	0.423±0.009	0.408±0.014
DeepHost [18]	0.885±0.017	0.870±0.023	0.398±0.104

Table S12: Comparing our graph model with other models and tools on phage host species prediction tasks.
The best performing model is in bold font, while the runner-up is underlined. Results are averaged over five random train-test splits.

Task	Model	Accuray	F1 score	AUC
Phage and ICE discrimination	with GCN	**0.934**	**0.899**	**0.981**
	without GCN	0.769	0.673	0.789
Phage integration site prediction	with GCN	**0.846**	**0.837**	**0.874**
	without GCN	0.823	0.809	0.867
Bacterial integration site prediction	with GCN	**0.810**	**0.789**	**0.855**
	without GCN	0.789	0.777	0.848
Phage lifestyle prediction	with GCN	**0.965**	**0.942**	**0.975**
	without GCN	0.941	0.903	0.966
		**Accuray**	**Weighted F1 score**	**Macro F1 score**
Phage host prediction	with GCN	**0.940**	**0.937**	**0.713**
	without GCN	0.821	0.787	0.549

Table S13: The performance comparison of models with and without graph convolutional layers. The best performance is bold.

		Accuray	F1 score	AUC
GP-GCN framework	Overall test sequences	0.934	0.899	0.981
	Unaligned test sequences	0.901	0.913	0.976
Without GCN module	Overall test sequences	0.903	0.832	0.971
	Unaligned test sequences	0.814	0.819	0.974
GP-GCN framework (d={0})	Overall test sequences	0.916	0.880	0.966
	Unaligned test sequences	0.878	0.872	0.962

Table S14: For the ablation study, we first align the test sequences to the training set with BLAST (e-value $< 10^{-5}$). Among the 606 test sequences, 253 sequences failed to align with the training set in this case, which are regarded as novel test sequences. Then we evaluate the performance of three models, the complete GP-GCN framework, the framework without the GCN module, and the framework with d={0}, on overall test sequences and novel test sequences.

Parameter type	Parameter	Parameter setting	Description
Model parameters	K	3	The length of k-mer.
	d_n	{0,1,2}	The gap lengths allowed in gapped patterns..
	node_hidden_dim	3	$\|h_v^{l+1}\|$.
	gcn_dim	128	$\|h_u^{l+1}\|$; The dimension of GCN embeddings.
	gcn_layer_num	2	The number of GCN layers.
	cnn_dim	64	The dimension of convolutional layer embeddings.
	cnn_layer_num	3	The number of convolutional layers.
	cnn_kernel_size	8	The kernel size of convolutional layers.
	fc_dim	100	The dimension of fully connected layer embeddings.
	dropout_rate	0.2	The dropout rate.
	pnode_nn	Yes	Whether to embed primary features into latent space.
	fnode_nn	Yes	Whether to embed target features into latent space.
Training parameters	learning_rate	1e-4	The learning rate for training.
	batch_size	64	The batch size for training.
	epoch_n	20	The number of training epochs.
	val_split	0.1	The validation set size.

Table S15: The default hyperparameters of the GP-GCN framework.

Task	Dataset	# of sequences	# of classifications	Accuracy
Species classification with MHC	IPD-MHC [20]	8,576	9	0.687
Regulatory item identification	hg38	30,000	3	0.839
lncRNA localization prediction	RNALocate [31]	769	4	0.664

Table S16: The statistics of the three extended applications for the GP-GCN framework.

Method	Accuracy	Weighted F1 score	Macro F1 score
GP-GCN (Ours)	**0.687±0.009**	**0.686±0.011**	**0.807±0.010**
AF-based	<u>0.663±0.017</u>	<u>0.663±0.017</u>	<u>0.776±0.008</u>
word2vec	0.514±0.027	0.493±0.044	0.651±0.024
Sequence-based*	0.599±0.028	0.604±0.026	0.724±0.032
Nucleotide Transformer	0.455±0.035	0.394±0.041	0.575±0.049
Gapped pattern frequency-based using			
k-Nearest Neighbors	0.656±0.015	0.649±0.016	0.756±0.017
Random Forest	0.662±0.015	<u>0.663±0.016</u>	0.775±0.015
MLP	0.628±0.017	0.634±0.018	0.771±0.014

Table S17: Comparing our graph model with other models on species classification with MHC task. The best performing model is in bold font, while the runner-up is underlined. Results are averaged over ten random train-test splits. * We set L to 2,000 according to the input length distribution (Figure S16).

Method	Accuracy	Weighted F1 score	Macro F1 score
GP-GCN (Ours)	**0.839±0.004**	**0.840±0.004**	**0.840±0.005**
AF-based	0.450±0.008	0.450±0.008	0.378±0.007
word2vec	<u>0.824±0.015</u>	0.818±0.004	<u>0.815±0.004</u>
Sequence-based*	0.672±0.007	0.673±0.007	0.673±0.007
Gapped pattern frequency-based using			
k-Nearest Neighbors	0.387±0.009	0.268±0.011	0.268±0.011
Random Forest	0.803±0.007	<u>0.805±0.007</u>	0.805±0.007
MLP	0.785±0.007	0.783±0.008	0.783±0.007

Table S18: Comparing our graph model with other models on regulatory item identification task. The best performing model is in bold font, while the runner-up is underlined. Results are averaged over ten random train-test splits. * We set L to 2,000 according to the input length distribution (Figure S16).

Method	Accuracy	Weighted F1 score	Macro F1 score
GP-GCN (Ours)	**0.664±0.017**	**0.656±0.019**	**0.566±0.005**
AF-based	0.457±0.064	0.457±0.064	0.362±0.081
word2vec	0.553±0.060	0.565±0.054	0.445±0.043
Sequence-based*	0.549±0.056	0.514±0.048	0.315±0.054
Gapped pattern frequency-based using			
k-Nearest Neighbors	0.438±0.045	0.302±0.042	0.165±0.009
Random Forest	<u>0.610±0.033</u>	0.559±0.024	0.384±0.060
MLP	0.602±0.033	<u>0.590±0.026</u>	0.435±0.024
GraphLncLoc	0.609±0.001	0.589±0.005	<u>0.502±0.009</u>

Table S19: Comparing our graph model with other models on lncRNA localization prediction task. The best performing model is in bold font, while the runner-up is underlined. Results are averaged over ten random train-test splits. * We set L to 10,000 according to the input length distribution (Figure S16).

References

[1] J Rodney Brister, Danso Ako-Adjei, Yiming Bao, and Olga Blinkova. NCBI viral genomes resource. *Nucleic Acids Research*, 43(D1):D571–D577, 2015.

[2] Dexi Bi, Zhen Xu, Ewan M Harrison, Cui Tai, Yiqing Wei, Xinyi He, Shiru Jia, Zixin Deng, Kumar Rajakumar, and Hong-Yu Ou. ICEberg: a web-based resource for integrative and conjugative elements found in Bacteria. *Nucleic Acids Research*, 40(D1):D621–D626, 2012.

[3] Luis F Camarillo-Guerrero, Alexandre Almeida, Guillermo Rangel-Pineros, Robert D Finn, and Trevor D Lawley. Massive expansion of human gut bacteriophage diversity. *Cell*, 184(4):1098–1109, 2021.

[4] Xiaofang Jiang, Andrew Brantley Hall, Ramnik J Xavier, and Eric J Alm. Comprehensive analysis of chromosomal mobile genetic elements in the gut microbiome reveals phylum-level niche-adaptive gene pools. *PloS One*, 14(12):e0223680, 2019.

[5] Torsten Seemann. Prokka: rapid prokaryotic genome annotation. *Bioinformatics*, 30(14):2068–2069, 2014.

[6] Xianglilan Zhang, Ruohan Wang, Xiangcheng Xie, Yunjia Hu, Jianping Wang, Qiang Sun, Xikang Feng, Shanwei Tong, Yujun Cui, Mengyao Wang, et al. Mining bacterial NGS data vastly expands the complete genomes of temperate phages. *bioRxiv*, 2021.

[7] Travis N Mavrich and Graham F Hatfull. Bacteriophage evolution differs by host, lifestyle and genome. *Nature Microbiology*, 2(9):1–9, 2017.

[8] Adam J Hockenberry and Claus O Wilke. BACPHLIP: Predicting bacteriophage lifestyle from conserved protein domains. *PeerJ*, 9:e11396, 2021.

[9] Arthur L Delcher, Kirsten A Bratke, Edwin C Powers, and Steven L Salzberg. Identifying bacterial genes and endosymbiont DNA with Glimmer. *Bioinformatics*, 23(6):673–679, 2007.

[10] L Steven Johnson, Sean R Eddy, and Elon Portugaly. Hidden Markov model speed heuristic and iterative HMM search procedure. *BMC Bioinformatics*, 11(1):1–8, 2010.

[11] Andrzej Zielezinski, Susana Vinga, Jonas Almeida, and Wojciech M Karlowski. Alignment-free sequence comparison: benefits, applications, and tools. *Genome Biology*, 18(1):1–17, 2017.

[12] Radim Řehůřek and Petr Sojka. Software Framework for Topic Modelling with Large Corpora. In *Proceedings of the LREC 2010 Workshop on New Challenges for NLP Frameworks*, pages 45–50, Valletta, Malta, May 2010. ELRA.

[13] Hugo Dalla-Torre, Liam Gonzalez, Javier Mendoza-Revilla, Nicolas Lopez Carranza, Adam Henryk Grzywaczewski, Francesco Oteri, Christian Dallago, Evan Trop, Bernardo P de Almeida, Hassan Sirelkhatim, et al. The nucleotide transformer: Building and evaluating robust foundation models for human genomics. *bioRxiv*, pages 2023–01, 2023.

[14] Yanrong Ji, Zhihan Zhou, Han Liu, and Ramana V Davuluri. Dnabert: pre-trained bidirectional encoder representations from transformers model for dna-language in genome. *Bioinformatics*, 37(15):2112–2120, 2021.

[15] Shufang Wu, Zhencheng Fang, Jie Tan, Mo Li, Chunhui Wang, Qian Guo, Congmin Xu, Xiaoqing Jiang, and Huaiqiu Zhu. Deephage: distinguishing virulent and temperate phage-derived sequences in metavirome data with a deep learning approach. *GigaScience*, 10(9):giab056, 2021.

[16] Julia Villarroel, Kortine Annina Kleinheinz, Vanessa Isabell Jurtz, Henrike Zschach, Ole Lund, Morten Nielsen, and Mette Voldby Larsen. HostPhinder: a phage host prediction tool. *Viruses*, 8(5):116, 2016.

[17] Clovis Galiez, Matthias Siebert, François Enault, Jonathan Vincent, and Johannes Söding. WIsH: who is the host? Predicting prokaryotic hosts from metagenomic phage contigs. *Bioinformatics*, 33(19):3113–3114, 2017.

[18] Ruohan Wang, Xianglilan Zhang, Jianping Wang, and Shuai Cheng Li. DeepHost: phage host prediction with convolutional neural network. *Briefings in Bioinformatics*, 23(1):bbab385, 2022.

[19] Jacques Neefjes, Marlieke LM Jongsma, Petra Paul, and Oddmund Bakke. Towards a systems understanding of MHC class I and MHC class II antigen presentation. *Nature reviews immunology*, 11(12):823–836, 2011.

[20] Giuseppe Maccari, James Robinson, Keith Ballingall, Lisbeth A Guethlein, Unni Grimholt, Jim Kaufman, Chak-Sum Ho, Natasja G De Groot, Paul Flicek, Ronald E Bontrop, et al. IPD-MHC 2.0: an improved inter-species database for the study of the major histocompatibility complex. *Nucleic acids research*, 45(D1):D860–D864, 2017.

[21] Simone Sommer. The importance of immune gene variability (MHC) in evolutionary ecology and conservation. *Frontiers in zoology*, 2:1–18, 2005.

[22] Robin Andersson and Albin Sandelin. Determinants of enhancer and promoter activities of regulatory elements. *Nature Reviews Genetics*, 21(2):71–87, 2020.

[23] Alan P Boyle, Sean Davis, Hennady P Shulha, Paul Meltzer, Elliott H Margulies, Zhiping Weng, Terrence S Furey, and Gregory E Crawford. High-resolution mapping and characterization of open chromatin across the genome. *Cell*, 132(2):311–322, 2008.

[24] Joana Carlevaro-Fita and Rory Johnson. Global positioning system: understanding long noncoding RNAs through subcellular localization. *Molecular cell*, 73(5):869–883, 2019.

[25] Min Li, Baoying Zhao, Rui Yin, Chengqian Lu, Fei Guo, and Min Zeng. GraphLncLoc: long non-coding RNA subcellular localization prediction using graph convolutional networks based on sequence to graph transformation. *Briefings in Bioinformatics*, 24(1):bbac565, 2023.

[26] Tianyu Cui, Yiying Dou, Puwen Tan, Zhen Ni, Tianyuan Liu, DuoLin Wang, Yan Huang, Kaican Cai, Xiaoyang Zhao, Dong Xu, et al. RNALocate v2. 0: an updated resource for RNA subcellular localization with increased coverage and annotation. *Nucleic acids research*, 50(D1):D333–D339, 2022.

[27] Ana Brandão, Diana P Pires, Lucas Coppens, Marleen Voet, Rob Lavigne, and Joana Azeredo. Differential transcription profiling of the phage LUZ19 infection process in different growth media. *RNA biology*, 18(11):1778–1790, 2021.

[28] Chi-Dung Yang, Yen-Hua Chen, Hsi-Yuan Huang, Hsien-Da Huang, and Ching-Ping Tseng. CRP represses the CRISPR/Cas system in E scherichia coli: evidence that endogenous CRISPR spacers impede phage P 1 replication. *Molecular Microbiology*, 92(5):1072–1091, 2014.

[29] Laura Mojardín and Margarita Salas. Global transcriptional analysis of virus-host interactions between phage ϕ29 and bacillus subtilis. *Journal of virology*, 90(20):9293–9304, 2016.

[30] E Fidelma Boyd, Brigid M Davis, and Bianca Hochhut. Bacteriophage–bacteriophage interactions in the evolution of pathogenic bacteria. *Trends in microbiology*, 9(3):137–144, 2001.

[31] Ting Zhang, Puwen Tan, Liqiang Wang, Nana Jin, Yana Li, Lin Zhang, Huan Yang, Zhenyu Hu, Lining Zhang, Chunyu Hu, et al. RNALocate: a resource for RNA subcellular localizations. *Nucleic acids research*, 45(D1):D135–D138, 2017.
